# A major locus controls local adaptation and adaptive life history variation in a perennial plant

**DOI:** 10.1186/s13059-018-1444-y

**Published:** 2018-06-04

**Authors:** Jing Wang, Jihua Ding, Biyue Tan, Kathryn M. Robinson, Ingrid H. Michelson, Anna Johansson, Björn Nystedt, Douglas G. Scofield, Ove Nilsson, Stefan Jansson, Nathaniel R. Street, Pär K. Ingvarsson

**Affiliations:** 1 Umeå Plant Science Centre, Department of Ecology and Environmental Science, Umeå University, 90187 Umeå, Sweden; 2 Centre for Integrative Genetics, Department of Animal and Aquacultural Sciences, Faculty of Life Sciences, Norwegian University of Life Sciences, PO Box 5003, Ås, Norway; 3 Umeå Plant Science Centre, Department of Forest Genetics and Plant Physiology, Swedish University of Agricultural Sciences, 901 83 Umeå, Sweden; 4 Stora Enso Biomaterials, 13104 Nacka, Sweden; 5 Umeå Plant Science Centre, Department of Plant Physiology, Umeå University, 90187 Umeå, Sweden; 6 Wallenberg Advanced Bioinformatics Infrastructure, Science for Life Laboratory, Uppsala University, Uppsala, Sweden; 7 Department of Ecology and Genetics, Evolutionary Biology, Uppsala University, Uppsala, Sweden; 8 Uppsala Multidisciplinary Center for Advanced Computational Science, Uppsala University, Uppsala, Sweden; 9 Present address: Department of Plant Biology, Uppsala BioCenter, Swedish University of Agricultural Sciences, PO Box 7080, 750 07 Uppsala, Sweden

**Keywords:** *Populus tremula*, Local adaptation, Genomic basis, *PtFT2*, Adaptive traits, Selective sweep

## Abstract

**Background:**

The initiation of growth cessation and dormancy represent critical life-history trade-offs between survival and growth and have important fitness effects in perennial plants. Such adaptive life-history traits often show strong local adaptation along environmental gradients but, despite their importance, the genetic architecture of these traits remains poorly understood.

**Results:**

We integrate whole genome re-sequencing with environmental and phenotypic data from common garden experiments to investigate the genomic basis of local adaptation across a latitudinal gradient in European aspen (*Populus tremula*). A single genomic region containing the *PtFT2* gene mediates local adaptation in the timing of bud set and explains 65% of the observed genetic variation in bud set. This locus is the likely target of a recent selective sweep that originated right before or during colonization of northern Scandinavia following the last glaciation. Field and greenhouse experiments confirm that variation in *PtFT*2 gene expression affects the phenotypic variation in bud set that we observe in wild natural populations.

**Conclusions:**

Our results reveal a major effect locus that determines the timing of bud set and that has facilitated rapid adaptation to shorter growing seasons and colder climates in European aspen. The discovery of a single locus explaining a substantial fraction of the variation in a key life-history trait is remarkable, given that such traits are generally considered to be highly polygenic. These findings provide a dramatic illustration of how loci of large-effect for adaptive traits can arise and be maintained over large geographical scales in natural populations.

**Electronic supplementary material:**

The online version of this article (10.1186/s13059-018-1444-y) contains supplementary material, which is available to authorized users.

## Backgrounds

Most species are distributed over heterogeneous environments across their geographic range and spatially varying selection is known to induce adaptation to local environments [1]. Local adaptation thus provides an opportunity to study population genetic divergence in action [2]. Although the interaction between gene flow and natural selection is well studied from a theoretical point of view and makes a number of testable predictions [3], there are to date few empirical studies investigating how local adaptation is established and maintained at the molecular level in natural populations.

Many perennial plants, such as forest trees, have wide geographic distributions and are consequently exposed to a broad range of environmental conditions, making adaptation to diverse environmental and climate conditions crucial in these species [4–7]. Natural populations of these plants are often locally adapted and display pronounced geographic clines in phenotypic traits related to climatic adaptation even in the face of substantial gene flow [5, 6]. One of the most important traits mediating local adaptation is initiation of growth cessation at the end of the growing season, which represents a critical life history trade-off between survival and growth in most perennial plants [8, 9]. Local adaptation in phenology traits, such as growth cessation, is well documented at the phenotypic level in many long-lived perennial species [2, 6]. Compared to traditional model and crop species that are usually annuals, naturally inbred and have rich genomic resources available, the genomic and evolutionary research in long-lived, outcrossing perennial species is much more difficult to conduct, and the genetic architecture of adaptive traits in such species is therefore still rather poorly understood [5, 6].

Here we investigate the genomic signatures of local adaptation across a latitudinal gradient that limits the length of the growing season in European aspen (*Populus tremula*). *P. tremula* is a dioecious and obligately outbreeding tree species; both seeds and pollen are wind-dispersed and usually show weak population genetic structure [10, 11]. Despite low genetic differentiation at neutral molecular markers, local populations display strong adaptive differentiation in phenology traits, such as the timing of bud set and growth cessation, across the latitudinal gradient [10]. In this study, we integrate whole genome re-sequencing with field and greenhouse experiments to characterize the genome-wide architecture of local adaptation in *P. tremula*. Using a combination of approaches, we identify a single genomic region, centered on a *P. tremula* homolog of *FLOWERING LOCUS T*2 (*PtFT*2), that controls a substantial fraction of the naturally occurring genetic variation in the timing of bud set. The region displays multiple signs of a recent selective sweep that appears to have been restricted to the northern-most populations. Our results provide evidence of a major locus that has facilitated rapid adaptation to shorter growing seasons and colder climates following post-glacial colonization.

## Results

### Genome sequencing, polymorphism detection, and population structure

**Fig. 1:**
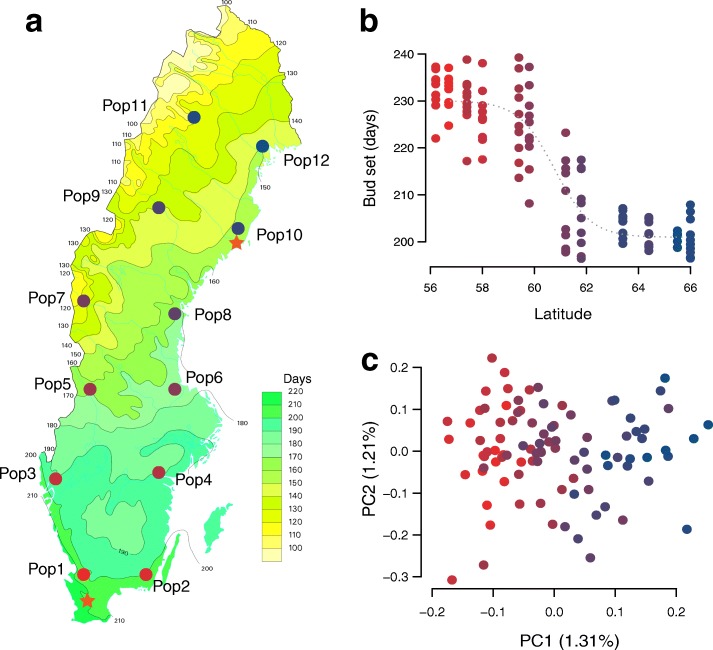
Geographic distribution and genetic structure of 94 aspen individuals. **a** Location of the 12 original sample sites of the SwAsp collection (*circles*) and the location of the two common garden sites (*orange stars*). The original collection sites span a latitudinal gradient of c. 10 latitude degrees across Sweden. **b** Genetic values for date of bud set for the 94 individuals included in the study across the two common gardens and three years (2005, 2006, and 2007). **c** Population structure in the SwAsp collection based on a PCA of 217,489 SNPs that were pruned to remove SNPs in high linkage disequilibrium (SNPs included all have *r*^2^ < 0.2). Although two axes are shown, only the first axis is significant (*P* = 3.65 × 10^−12^, Tacey-Widom test, 1.31% variance explained)

In this study, we used a total of 94 unrelated *P. tremula* trees that were originally collected from 12 sites spanning c. 10° of latitude (~ 56–66 °N) across Sweden (the SwAsp collection from [12], see also Additional file 1: Table S1). Earlier studies have shown that the SwAsp collection displays a strong latitudinal cline in the timing of bud set (Fig. 1a, b) [10–12]. We performed whole genome re-sequencing of all 94 aspens and obtained a total of 1139.2 Gb of sequence, with an average sequencing depth of ~ 30 × per individual covering > 88% of the reference genome (Additional file 1: Table S1). After stringent variant calling and filtering, we identified a total of 4,425,109 high-quality single nucleotide polymorphisms (SNPs) with a minor allele frequency (MAF) > 5%.

We found very weak population structure across the entire range using principal component analysis (PCA) [13], with a single significant axis separating individuals according to latitude (*r* = 0.889, *P* < 0.001) but explaining only 1.3% of the total genetic variance (Fig. 1c; Additional file 2: Table S2). Consistent with this, a Mantel test also showed a weak pattern of isolation by distance (IBD; *r* = 0.210; *P* = 0.047; Additional file 3: Figure S1). Swedish populations of *P. tremula* have gone through a recent admixture of divergent post-glacial lineages following the Last Glacial Maximum (LGM) [14] and it is possible that this is capable of generating a genome-wide pattern of clinal variation. However, extensive gene flow among populations of *P. tremula*, as suggested by the extremely low level of genome-wide population genetic differentiation (mean *F*_ST_ = 0.0021; Additional file 3: Figure S2), has almost eradicated any such signal across the genome.

### Identifying genomic variants associated with local adaptation

**Fig. 2:**
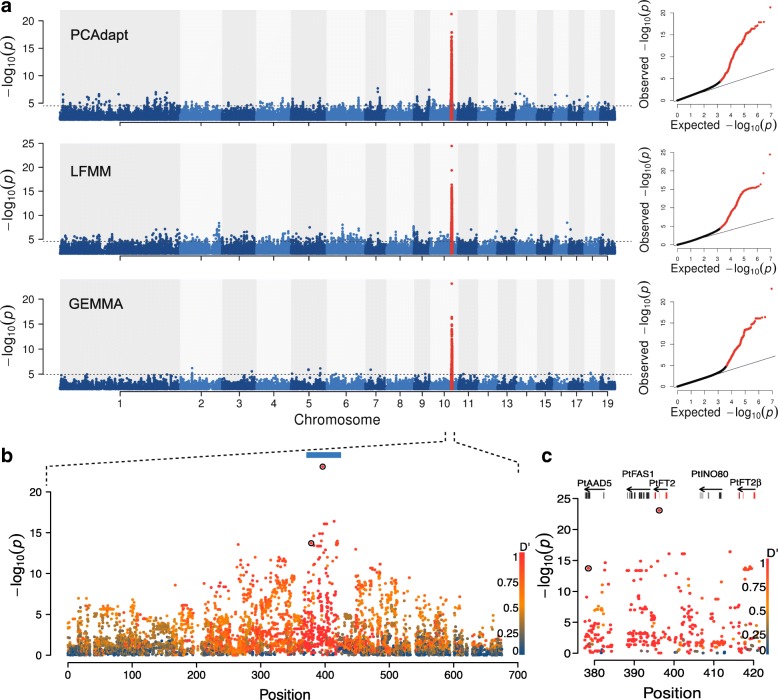
Local adaptation signals across the genome. **a** Manhattan plots for SNPs associated with population structure (PCAdapt), climate variation (LFMM), and phenotype (GEMMA). The 700-kbp region surrounding *PtFT2* gene (marked in *red*) is identified by all methods. The *dashed line* represents the significance threshold for each method. Quantile-quantile *plot* is displayed in the *right panel*, with significant SNPs highlighted in *red*. **b** Magnification of the phenotype association results (from GEMMA) for the region surrounding *PtFT2* on Chr10. The coordinates correspond to the region 16.3 Mbp-17.0 Mbp on Chr10. Individual data points are colored according to LD with the most strongly associated SNP (Potra001246:25256). The two potential causal variants identified by CAVIAR within this region are marked by *black circles*. **c** Close-up view of the phenotype association results (from GEMMA) in a region corresponding to the *blue bar* in (**b**). This region contains the two *PtFT2* homologs (*red* - exons, *blue* - UTRs) and several other genes (*dark gray* - exons, *light grey* - UTRs)

We used three complementary approaches to identify candidate SNPs involved in local adaptation. First, we identified SNPs that were most strongly associated with the observed population structure using PCAdapt [15]. Second, we identified SNPs showing strong associations with environmental variables based on a latent factor mixed-effect model (LFMM) [16]. Finally, we performed genome-wide association mapping (GWAS) on the timing of bud set, our target adaptive trait, using GEMMA (Fig. 2a, [16, 17]). SNPs identified as significant (false discovery rate [FDR] < 0.05) by the three methods showed a large degree of overlap (Additional file 3: Figure S3) and for subsequent analyses we consider SNPs that were identified as significant by at least two of the three methods to be involved in local adaptation. In total, 99.2% of the 910 SNPs identified by all three methods and 89.1% of the additional 705 SNPs identified by two methods were located in a single region spanning c. 700 kbp on chromosome 10 (Fig. 2a, b; Additional file 3: Figure S4; Additional file 4: Table S3).

SNPs associated with local adaptation displayed strong clinal patterns in allele frequencies with latitude, in stark contrast to 10,000 SNPs randomly selected from across the genome that displayed no or negligible differences among populations (Additional file 3: Figure S5). The 700-kbp region on chromosome 10 encompasses 92 genes and the most strongly associated variants for all three tests are located in a region containing two *P. tremula* homologs of the *Arabidopsis FLOWERING LOCUS T* (*PtFT2*; Potra001246g10694 and an unannotated copy located c. 20 kbp upstream of *PtFT*2, tentatively named *PtFT*2β) (Fig. 2b, c). *FT* is known to be involved in controlling seasonal phenology in perennial plants [18] and has previously been implicated in regulating short-day induced growth cessation, bud set, and dormancy induction in *Populus* [19, 20].

We observed that structure of the *PtFT2* locus is conserved across *Populus* species, but not between *Populus* and *Salix* (Additional file 3: Figure S6). Although both copies of *PtFT*2 appear to be expressed (Additional file 3: Figure S7), the SNP showing the strongest signal of local adaptation across all three methods (Potra001246:25256) was located in the third intron of the previously annotated copy of *PtFT2* (Potra001246g10694) (Fig. 2c). This SNP explain 65% of the observed genetic variation in the timing of bud set across years and sites. Furthermore, it was identified as having highest probability of being the causal variant within the 700-kbp region by CAVIAR [21] (Fig. 2b, c), a fine-mapping method that accounts for linkage disequilibrium (LD) and effect sizes to rank potential causal variants. Another potentially causal SNP (Potra001246:43095) in this region is in strong LD with Potra001246:25256 (Fig. 2c). Therefore, we identify *PtFT2* as a candidate gene, and henceforth, we refer to the entire ~ 700-kbp region centered on *PtFT2* as the *PtFT2* locus. We note, however, that this region potentially harbors many SNPs that could individually contribute to bud set and hence could be involved in local adaptation.

### Evidence of rapid adaptive evolution

**Fig. 3:**
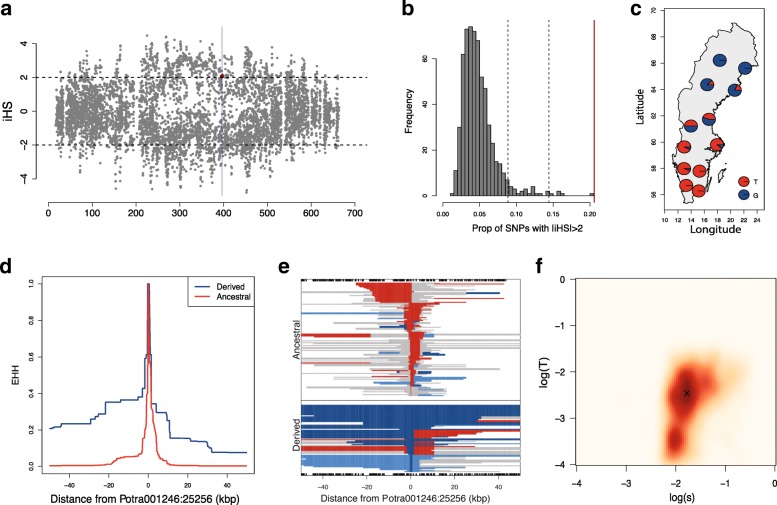
Evidence of positive selection centered on the *PtFT2* locus. **a** Patterns of normalized iHS scores (*y-axes*) across the ~ 700-kbp genomic region (*x-axis*) around the *PtFT2* gene (*vertical light gray bar*). The *dashed horizontal lines* indicate the threshold of positive selection signal (|iHS| > 2). The *red dot* indicates the SNP (Potra001246:25256) showing the strongest signal of local adaptation. **b** A high concentration of significant |iHS| signals was found in the ~ 700-kbp region surrounding *PtFT2* (marked as *red line*) compared to the genome-wide distribution (based on dividing the genome into non-overlapping windows of 700 kbp). The dashed lines represent the 95% and 99% quantiles, respectively. **c** Allele frequencies of the most strongly associated SNP Potra001246:25256 for the 12 original populations of the SwAsp collection. **d** The decay of extended haplotype homozygosity (EHH) of the derived (*blue*) and ancestral (*red*) alleles for the SNP Potra001246:25256. **e** The extent of the three most common haplotypes at Potra001246:25256. Rare recombinant haplotypes were pooled and are displayed in *gray*. **f** Joint inference of allele age and selection coefficient for the region surrounding *PtFT*2

In order to gain further insight into the evolutionary history of the *PtFT2* locus, we performed several haplotype-based tests to examine the presence of recent positive selection in this region. We calculated the standardized integrated haplotype score (iHS) [21, 22] for all SNPs (8570 SNPs where information of ancestral or derived states was available) located in the 700-kbp region (Fig. 3a). Positive selection signals, revealed by |iHS| > 2.0, were observed for 20.6% of all tested SNPs. We found that the region surrounding *PtFT2* contained the highest concentration of significant hits by the iHS test across the genome (Fig. 3b), confirming that *PtFT2* locus as the strongest candidate for positive selection in the Swedish populations of *P. tremula*. Similar results were found when the number of segregating sites by length (nSL) [23], which has proven sensitive for detecting incomplete selective sweeps, was calculated for these same loci (Additional file 3: Figure S8). We further performed the extended haplotype homozygosity (EHH) test [24], centering on the most strongly associated SNP (Potra001246:25256), to explore the extent of haplotype homozygosity around the selected region. The core haplotype carrying the derived allele (G) had elevated EHH and exhibited long-range LD relative to haplotypes carrying the ancestral allele (T) (Fig. 3d). Also, haplotypes carrying the derived allele were longer than those carrying the ancestral allele (Fig. 3e). Notably, the derived allele with high EHH is largely restricted to the four high-latitude populations and almost absent in the southern-most populations (Fig. 3c), implying that *PtFT2* locus has likely been subjected to geographically restricted selective sweeps [25].

**Fig. 4:**
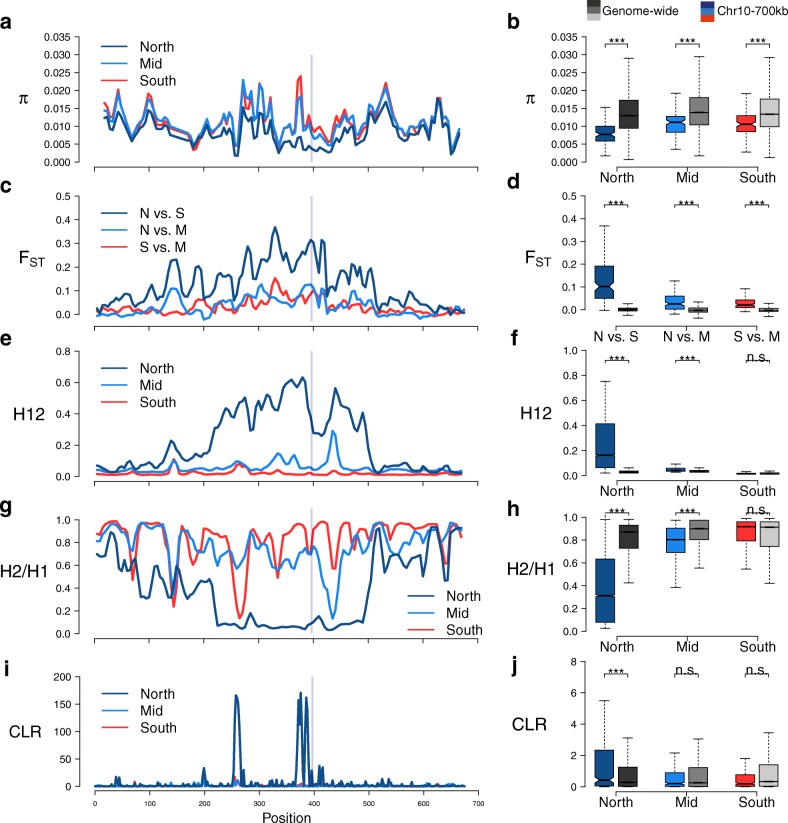
Geographically restricted selective sweep in northern-most populations. *Left panels*: A magnified view of different summary statistics that are sensitive to the effects of a selective sweep for the ~ 700 kbp region surrounding *PtFT2*. The *gray bar* marks the location of the *PtFT*2 gene. *Right panels*: Comparison of these statistics between the *PtFT2* region (*colored boxplot*) and the genome-wide averages (*gray boxplot*). Statistics were calculated separately for individuals from southern (population 1–6), middle (populations 7–8), and northern (populations 9–12) in Sweden. **a**, **b** Nucleotide diversity, π. **c**, **d** Genetic differentiation, *F*_ST_. **e**, **f** H12. **g**, **h** H2/H1. **i**, **j** Composite likelihood ratio (CLR) test for the presence of a selective sweep

To further understand the evolution of functional differences between northern and southern *PtFT2* alleles, we examined the patterns of genetic variation at the *PtFT2* locus separately for South (pop 1–6), Mid (pop 7–8), and North (pop 9–12) populations. First, we found that the nucleotide diversity at the *PtFT2* locus was significantly below the genome-wide averages in all groups of populations (Fig. 4a, b; Additional file 5: Table S4), which was consistent with the expectation of a strong selective event [26]. In particular, northern populations were observed to have a much stronger reduction of genetic diversity relative to other populations (Fig. 4a, b). Additionally, the level of genetic differentiation among populations was exceptionally high at *PtFT2* locus compared with genomic background, especially between southern and northern populations (Fig. 4c, d; Additional file 5: Table S4), implying that spatially varying selection has likely driven latitudinal differentiation at this locus. Furthermore, high H12 but low H2/H1 statistics [27] was only observed in northern populations (Fig. 4e–h; Additional file 5: Table S4), providing a clear indication of a single adaptive haplotype that has risen to high frequency among these populations (Additional file 3: Figure S9). Finally, we performed a composite-likelihood based (CLR) test and separately evaluated the evidence of positive selection in different groups of populations. As expected for positive selection, a distorted site frequency spectrum with an excess of rare and high frequency derived variants near the *PtFT2* locus was only found in northern populations (Fig. 4i, j; Additional file 5: Table S4). Overall, all these findings provide compelling evidence for the occurrence of a strong selection on a single variant at the *PtFT2* locus in the northern-most Swedish populations of *P. tremula*.

The observation of a single adaptive haplotype rising to high frequency in high-latitude populations (Fig. 4; Additional file 3: Figure S9) is consistent with a selective sweep pattern, where adaptation can result either from a de novo mutation or from a low frequency standing variant that was already present in the population before the onset of selection [28]. Assuming the causal mutation appeared near the time of the onset of selection, we used an Approximate Bayesian Computation (ABC) method [29] to estimate jointly the age and strength of selection acting on the northern allele. The results (Fig. 3f) point to a recent origin of the northern allele (*T* = 18,952 years, 95% credible interval = 719–114,122 years) and that selection during the sweep has been relatively strong (s = 0.016, 95% credible interval = 0.006–0.192). This suggests that the adaptive event that occurred in northern-most populations of *P. tremula* most likely represents an evolutionary response to the harsher environmental conditions experienced by these populations during the post-glacial colonization of northern Scandinavia.

### PtFT2 regulates the timing of bud set

**Fig. 5:**
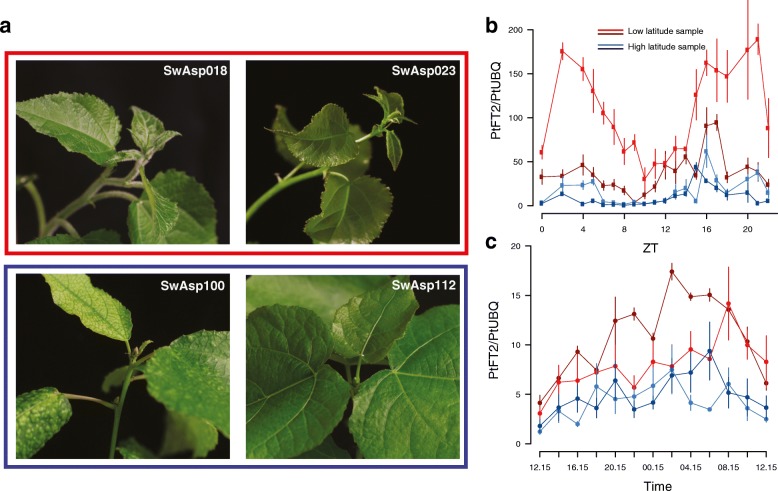
*PtFT2* expression affects short-day induced growth cessation and bud set in *P. tremula*. **a** Bud set phenotype under 19-h day-length conditions. Two southern clones (marked with a *red box*, SwAsp 018, Ronneby, latitude 56.2 °N; SwAsp 023, Vårgårda, latitudes 58 °N) and two northern clones (marked with a *blue box*, SwAsp 100, Umeå, latitude 63.9 °N; SwAsp 112, Luleå, latitudes 65.7 °N) were chosen to be analyzed. Trees were grown under 23-h day length for one month and then shifted to 19-h day length. Photos were taken one month after the shift to 19-h day length. **b** Dynamic expression analysis of *PtFT2* in two southern clones (*red*, SwAsp018 and SwAsp023) and two northern clones (*blue*, SwAsp100 and SwAsp112) from the greenhouse experiment. The genotypes of these trees at the most strongly associated *PtFT*2 SNP are SwAsp018: T/T, SwAsp023: T/T, SwAsp100: not available and SwAsp 112: G/G. Samples for RT-PCR were taken two weeks after the trees were shifted to 19-h day length. *Error bars*, ±standard deviation. ZT zeitgeber time. **c** Dynamic expression analysis of *PtFT2* in two southern clones (*red*, SwAsp005 and SwAsp023) and two northern clones (*blue*, SwAsp100 and SwAsp116) from common garden experiment. The genotypes of these trees at the most strongly associated *PtFT*2 SNP are SwAsp005: T/T, SwAsp023: T/T, SwAsp100: not available and SwAsp 112: G/G. Samples were collected in the Sävar common garden in early July 2014

Although the extensive LD in the immediate vicinity of the *PtFT*2 locus (Fig. 2b) makes it hard to identify the true causal SNP(s) that are involved in mediating natural variation in bud set, we found that the significantly associated SNPs are overall enriched in non-coding regions located in and around genes and show a deficit in intergenic regions (Additional file 3: Figure S10; Additional file 4: Table S3). One possible way that functional variation is mediated by these SNPs is thus by altering expression patterns of related genes across the latitudinal gradient. To further assess the possibility that patterns of *PtFT*2 expression is involved in mediating local adaptation, we selected two southern genotypes and two northern genotypes for greenhouse and field experiments in order to test whether *PtFT2* expression regulates the timing of growth cessation and bud set. In greenhouse experiments, we found that the two northern genotypes showed rapid growth cessation and bud set following a shift from long (23-h day length) to short day (19-h day length) conditions whereas the two southern genotypes continued active growth under the same conditions (Fig. 5a). Analyses of *PtFT*2 gene expression in these genotypes show a strong downregulation of *PtFT*2 in the northern genotypes in conjunction with growth cessation and bud set (Fig. 5b; Additional file 6: Table S5). Similarly, under field conditions we observe that northern genotypes also show lower expression of *PtFT*2 even at a time point when all genotypes were actively growing (Fig. 5c).

**Fig. 6:**
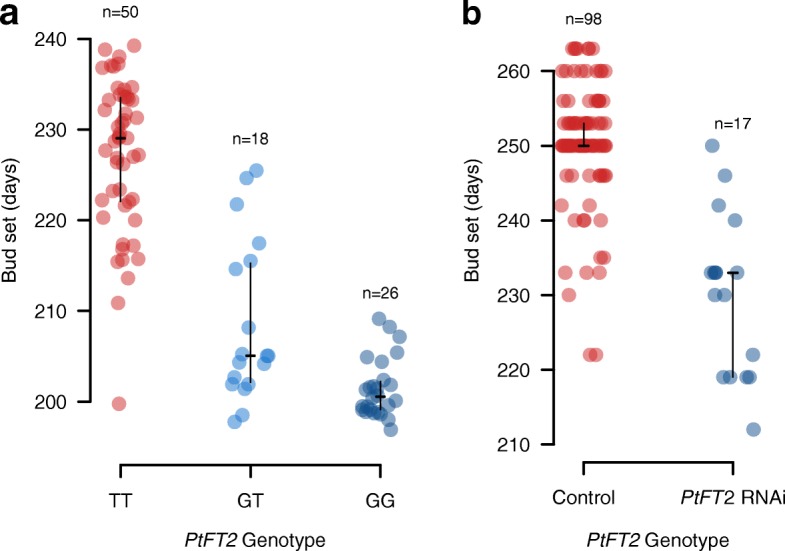
Phenotypic effects of *PtFT2*. **a** The timing of bud set for the three genotypes classes at the *PtFT2* SNP (Potra001246:25256) that displays the strongest signal of local adaptation identified by all three methods as shown in Fig. 2a. The *plot* displays mean genotype bud set after correcting for common garden site, year, and block effects. The *horizontal line* indicates the median value and the *vertical line* marks the interquartile range. The number of genotypes in the respective classes is indicated above the figure. **b** The timing of bud set for wild type control lines and transgenic *PtFT2* lines in the field experiments at Våxtorp. The structure of the plots is the same as in (**a**)

Furthermore, downregulation of the *PtFT*2 expression using RNA interference (RNAi) to approximately 20% of wild-type levels accelerates bud set by c. 23 days, a difference that is comparable to the differences we observe between the most extreme phenotypes in our field-collected trees (Fig. 6). For instance, wild-collected trees carrying the derived G allele in homozygous form for the most strongly associated SNP in *PtFT2* (Potra001246:25256) set bud on average 28 days earlier than those homozygous for the ancestral T allele, with the derived G allele showing partial dominance (Fig. 6a). The RNAi experiment thus provides additional evidence that differences in gene expression of *PtFT2* are involved in mediating the phenotypic differences we observe in bud set between northern and southern genotypes.

## Discussion

To date, only a small number of candidate genes have been used to identify potential loci linked to traits involved in local adaptation in *P. tremula* [11, 30, 31]. Here we have substantially expanded our earlier studies by utilizing data from whole genome re-sequencing to local environmental variables and phenotypic variation in a key adaptive life-history trait in order to investigate the genomic basis of local adaptation in *P. tremula*. We identify a locus, centered on *PtFT*2, that has a major effect on phenotypic variation in bud set and that has played a key role in the establishment of local adaptation of *P. tremula*. The likely target of the selective sweep, *PtFT2*, is a *P. tremula* homolog of the *Arabidopsis FT* gene that plays a central and widely conserved role in day-length perception and seasonal regulation of photoperiodic responses [32]. In *Populus*, the *FT* gene is represented by two functionally diverged paralogs where *PtFT*1 has been speculated to retain the function of reproductive initiation whereas *PtFT*2 acts to maintain growth and prevent bud set [18, 19]. We observe that differences in *PtFT*2 gene expression between genotypes from southern and northern Swedish populations are associated with the timing of bud set in response to variable day lengths in different environments (Fig. 5b, c). Transgenic downregulation of *PtFT*2, under field conditions, yields a phenotype that closely mimics variation found in our wild collected trees, further implying that non-coding regulatory variation in or around *PtFT*2 mediate local adaptation in bud set by altering the level and timing of *PtFT*2 expression. Moreover, a study in the related species *Populus trichocarpa* also identified an association between a non-coding variant at *PtFT2*, a SNP in the second intron, and naturally occurring variation in bud set [20]. Although the exact causal mutations differ, this demonstrates that parallel adaptive changes in the timing of bud set between *P. tremula* and *P. trichocarpa*, two species that diverged more than 7 million years ago and that occur on different continents, has involved changes in the same orthologous gene.

While *PtFT2* has been shown to contribute to local adaptation in Swedish populations of *P. tremula*, we only observe a signal of a strong and recent selective sweep at this locus in the four northern-most populations. This selective event has likely been driven by adaptation in response to the substantially shorter growing seasons that *P. tremula* has encountered at northern latitudes during the post-glacial colonization of northern Scandinavia following the last glaciation. One caveat concerning the selective scans performed in this study is that splitting populations into groups along a geographic transect (i.e. latitude) could confound inference of the underlying selective and demographic forces. For instance, it is possible that adaptation to spatially varying selection in Swedish populations of *P. tremula* have arisen in response to continuous rather than discrete environment clines [10]. In addition, the estimated age of the adaptive mutation at the *PtFT2* locus coincides with recent post-glacial re-colonization of northern Scandinavia and it is thus possible that strong genetic drift at the front of the range expansion have promoted surfing of the adaptive allele in the newly colonized regions [33, 34].

The weak population genetic structure we observe in our samples, combined with the fact that both pollen and seeds are wind dispersed in *P. tremula*, suggest that gene flow among Swedish populations of *P. tremula* is likely relatively high. In accordance with recent theoretical predictions [3], our findings show that despite the relatively high, inferred rates of gene flow, strong selection for local adaptation is acting to maintain the large-effect beneficial alleles that underlie the locally adaptive traits. Compared to small-effect loci that are prone to swamping and only transiently contribute to local adaptation [3, 35], large-effect loci are more likely to establish and persist over longer time scales as they are able to resist the homogenizing effect of migration [3]. The distribution of number and effect size for variants controlling adaptive traits is therefore expected to shift to few large-effect loci under persistent migration-selection balance [3] compared with models from isolated populations [36]. Multiple mechanisms can give rise to the characteristic pattern in *P. tremula* where a single locus explains most of the variation for a key life-history trait and facilitates rapid adaptation. First, the presence of genomic rearrangements, such as chromosomal inversions, that suppress recombination can be favored by natural selection and cause the clustering of SNPs associated with local adaptation at the *PtFT2* locus [37, 38]. However, in contrast to expectations from the presence of an inversion, we did not observe blocks of elevated LD around the *PtFT2* locus (Additional file 3: Figure S11). LD in this region decays rapidly and falls to background levels within a few thousand bases, similar to what is seen in other regions genome-wide (Additional file 3: Figure S11a). This indicates that frequent recombination has occurred in this region and that the clustering of SNPs involved in local adaptation most likely arose from a selective sweep instead of an inversion [39]. Nonetheless, owing to the limited ability to detect inversions using short-insert paired reads, future characterization of structural variation across the genome is clearly required to determine whether genomic rearrangements are involved in mediating signals of adaptation in the *Populus* genome. Second, the establishment probability of additional adaptive mutations can be increased in the vicinity of a locus undergoing strong divergent selection, leading to a genomic architecture where multiple, tightly linked loci are controlling an adaptive trait [39, 40]. However, recent theoretical work has shown that the conditions for such establishment of de novo linked beneficial mutations are rather restrictive [41]. Instead, another potentially more important mechanism for the formation of “genomic islands” of strong genetic differentiation is via secondary contact and the erosion of pre-existing genetic divergence, which is a process that can be very rapid, especially compared to the alternative scenario that involves the fixation of novel mutations [41]. This mechanism provides a tantalizing hypothesis for *P. tremula* where earlier studies have established the existence of a hybrid zone between divergent post-glacial lineages in Scandinavia [14, 41]. The selective sweep at *PtFT*2 is geographically restricted and likely occurred before secondary contact. Therefore, the large genomic “island” of divergence that we observe surrounding the *PtFT*2 locus is a strong candidate for having evolved via erosion following secondary contact.

## Conclusions

Our study identifies a single genomic region containing the *PtFT2* gene that has a major effect on regulating the timing of bud set and that has facilitated rapid local adaptation in *P. tremula* across a latitudinal gradient in Sweden. Natural selection is actively maintaining alternate alleles at this locus despite low genetic differentiation across the rest of the genome. In particular, we identify a strong and recent selective sweep that is restricted to the northern-most populations. This adaptation has thus likely arisen and been driven to fixation during the post-glacial colonization of northern Scandinavia in response to the substantially shorter growing seasons that are characteristic of northern latitudes.

Although the *FT* gene has repeatedly gone through duplications and functional diversifications in many plants, variation within and around these *FT*-like genes are involved in mediating adaptive responses to photoperiod changes and altering overall fitness in a wide range of plant species [42]. Given the central role of *FT* as a key integrator of diverse environmental signals [32], it is perhaps not surprising that *FT* is more likely to act like an evolutionary hotspot for rapid adaptation to changing environmental conditions compared to other genes in the photoperiodic pathway (Additional file 3: Figure S12) and that these adaptations are mediated through *cis*-regulatory changes [43, 44]. *FT* thus appears to serve as evolutionary “master switch” for adaptive life-history variation, similar to what have been seen for a few other loci in plants, such as *FLC* [45], *FRI* [46], and *DOG*1 [47, 48].

## Methods

### Sample collection and sequencing

We collected material from all available trees in the Swedish Aspen (SwAsp), which consists of 116 individuals collected from 12 different locations spanning the distribution range in Sweden [12] (Fig. 1a). Leaf material was sampled from one clonal replicate of each individual growing at a common garden experiment located in Sävar, northern Sweden. Total genomic DNA for each individual was extracted from frozen leaf tissue using the DNeasy plant mini prep kit (QIAGEN, Valencia, CA, USA). Paired-end sequencing libraries with an average insert size of 650 bp were constructed for all samples according to the Illumina manufacturer’s instructions. Whole genome sequencing and base calling were performed on the Illumina HiSeq 2000 platform for all individuals to a mean, per-sample depth of approximately 30× at the Science for Life Laboratory, Stockholm, Sweden.

### Sequence quality checking, read mapping, and post-mapping filtering

A total of 103 SwAsp individuals were successfully sequenced. Before read mapping, we used Trimmomatic v0.30 [49] to identify reads with adapter contamination and to trim adapter sequences from reads. After checking the quality of the raw sequencing data using FastQC (https://www.bioinformatics.babraham.ac.uk/projects/fastqc/), the quality of sequencing reads was found to drop towards the ends of reads (Additional file 3: Figure S13). We therefore used Trimmomatic v0.30 to trim bases from both ends of the reads if their qualities were < 20. Reads < 36 bases after trimming were discarded completely.

After quality control, all high-quality reads were mapped to a de novo assembly of the *P. tremula* genome (available at http://popgenie.org; [50]) using the BWA-MEM algorithm with default parameters using bwa-0.7.10 [51]. We used MarkDuplicates methods from the Picard packages (http://broadinstitute.github.io/picard/) to correct for the artifacts of PCR duplication by only keeping one read or read-pair with the highest summed base quality among those of identical external coordinates and/or same insert lengths. Alignments of all paired-end and single-end reads for each sample were then merged using SAMtools 0.1.19 [52]. Sequencing reads in the vicinity of insertions and deletions (indels) were globally realigned using the RealignerTargetCreator and IndelRealigner in the Genome Analysis Toolkit (GATK v3.2.2) [53]. To minimize the influence of mapping bias, we further discarded the following site types: (1) sites with extremely low (< 400× across all samples, i.e. less than an average of 4× per sample) or extremely high coverage (> 4500×, or approximately twice the mean depth at variant sites) across all samples after investigating the coverage distribution empirically; (2) sites with a high number of reads (> 200×, that is on average > 2 reads per sample) with mapping score equaling zero; (3) sites located within repetitive sequences as identified using RepeatMasker [54]; (4) sites that were in genomic scaffolds with a length < 2 kbp.

### SNP and genotype calling

SNP calling in each sample was performed using the GATK HaplotypeCaller and GenotypeGVCFs were then used to perform the multi-sample joint aggregation, re-genotyping, and re-annotation of the newly merged records among all samples. We performed several filtering steps to minimize SNP calling bias and to retain only high-quality SNPs: (1) remove SNPs at sites not passing all previous filtering criteria; (2) retain only bi-allelic SNPs with a distance of > 5 bp away from any indels; (3) remove SNPs for which the available information derived from < 70% of the sampled individuals after treating genotypes with quality score (GQ) < 10 as missing; (4) remove SNPs with an excess of heterozygotes and deviates from Hardy–Weinberg equilibrium test (*P* value < 1e-8). After all steps of filtering, a total of 4,425,109 SNPs with minor allele frequency > 5% were left for downstream analysis. Finally, the effect of each SNP was annotated using SnpEff version 3.6 [55] based on gene models from the *P. tremula* reference genome (available at http://popgenie.org); the most deleterious effect was selected if multiple effects occurred for the same SNP using a custom Perl script.

### Relatedness, population structure, and isolation by distance

To identify closely related individuals and to infer population structure among the sampled individuals, we discarded SNPs with missing rate > 10%, MAF < 5%, and that failed the Hardy–Weinberg equilibrium test (*P* < 1 × 10^−6^) after all filtering steps as shown above. We also generated LD-trimmed SNP sets by removing one SNP from each pair of SNPs when the correlation coefficients (*r*^2^) between SNPs exceed 0.2 in blocks of 50 SNPs using PLINK v1.9 [56]. This yielded 217,489 independent SNPs that were retained for downstream analyses of population structure. First, we used PLINK v1.9 to estimate identity-by-state (IBS) scores among pairs of all individuals. Nine individuals were excluded from further analyses due to their high pairwise genetic similarity with another sampled individual (IBS > 0.8), leaving a total of 94 “unrelated” individuals for all subsequent analyses (Additional file 3: Figure S14). Then, we used the smartpca program in EIGENSOFT v5.0 [13] to perform the PCA on the reduced set of genome-wide independent SNPs. A Tracey-Widom test, implemented in the program twstats in EIGENSOFT v5.0, was used to determine the significance level of the eigenvectors. Finally, IBD analysis was computed based on the pairwise comparison of the genetic and geographic distances between populations. We calculated the population differentiation coefficient (*F*_ST_) [57] for each pair of the 12 populations using VCFtools v0.1.12b [58]. The relationship between genetic distance measured as *F*_ST_/(1-*F*_ST_) and geographic distance (km) was evaluated using Mantel tests in the R package “vegan” [59]; the significance of the correlation was estimated based on 9999 permutations.

### Screening for SNPs associated with local adaptation

We used three conceptually different approaches to test for genome-wide signatures of local adaptation. First, we detected candidate SNPs involved in local adaptation using the PCA as implemented in PCAdapt [60]. PCAdapt examines the correlations (measured as the squared loadings *ρ*^2^_jk_, which is the squared correlation between the *j*th SNP and the *k*th principal component [PC]) between genetic variants and specific PCs without any prior definition of populations. As only the first PC was significant from the PCA (see “Results”), we only estimated the squared loadings *ρ*^2^_j1_ with PC1 to identify SNPs involved in local adaptation. Our results showed that most outlier SNPs that were highly correlated with the first population structure PC also had high *F*_ST_ values between populations (Additional file 3: Figure S15). Assuming a chi-square distribution (degree of freedom = 1) for the squared loadings *ρ*^2^_j1_, as suggested by [60], we used PCAdapt to compute *P* values for all SNPs and then calculated the FDR using the method of Storey and Tibshirani [61] to generate a list of candidate SNPs showing significant associations to population structure. Only SNPs with FDR < 5% were retained as those significantly involved in local adaptation.

Second, we tested for the presence of candidate SNPs that exhibited high correlations with environmental gradients. To do this, a total of 39 environmental variables were analyzed (Additional file 7: Table S6). Precipitation and temperature values were retrieved from WorldClim version 1 [62]. Sunshine hours, photosynthetically active radiation, and ultraviolet (UV) radiation were obtained using the STRÅNG data model at the Swedish Meteorological and Hydrological Institute (SMHI) (http://strang.smhi.se). Values were collected from the years 2002–2012 for the original sample coordinates of each SwAsp individual and the average values over years were then calculated. The environmental variables include latitude, longitude, altitude, the number of days with temperatures > 5 °C, UV irradiance, the photosynthetic photon flux density (PPFD), sunshine duration, monthly and annual average precipitation, and temperature. Due to the high degree of correlation among these environmental variables (Additional file 3: Figure S16a), we performed a PCA on these variables using the “prcomp” function in R to identify PCs that best summarized the range of environmental variation. The first environmental PC, which explained > 60% of the total variance (Additional file 3: Figure S16b,c) and had the strongest loadings for the length of growing season (Additional file 3: Figure S16d), was kept to represent our target environmental variable for further analyses. We then used a latent factor mixed-effect model (LFMM) implemented in the package LEA in R [63] to investigate associations between SNPs and the first environmental PC while simultaneously accounting for population structure by introducing unobserved latent factors into the model [16]. Due to the weak population structure found in the SwAsp collection (see “Results”), we ran the LEA function *lfmm* with the number of latent factors (*K*) in the range of 1–3, using 5000 iterations as burn-in followed by 10,000 iterations to compute LFMM parameters for all SNPs. This was performed five times for each value of *K*; we observed identical results across both different values of *K* and across independent runs within each value of *K* (data not shown). We only showed the results using *K* = 2 to account for the background population structure. LFMM outliers were detected as those SNPs with FDR < 0.05 after using the method of Storey and Tibshirani [61] to account for multiple testing.

Third, we obtained previously published measurements of the timing of bud set, which is a highly heritable trait that shows strong adaptive differentiation along the latitudinal gradient [31]. To measure phenotypic traits, all SwAsp individuals have previously been clonally replicated (four ramets per individual) and planted at two common garden sites in 2004 (Sävar, 63 °N, and Ekebo, 56 °N) (Fig. 1a). The common garden set-up is described in detail in Luquez et al. [12]. The timing of bud set was scored twice weekly starting from mid-July and continuing until all trees had set terminal buds. Bud set measurements were scored in three consecutive years, 2005–2007, in both common gardens [10]. A severe drought in Sävar caused most of the trees to set bud prematurely in 2006 and we therefore excluded data from Sävar in 2006 in all downstream analyses (see Ingvarsson et al. [31] for further discussion). We combined data on bud set from the two common garden sites and years by predicting genetic values with best linear unbiased prediction (BLUP) for all individuals. ASReml [64] was used to fit Eq. 1 to the data for calculating BLUP using restricted maximum-likelihood techniques:

1 $$ {\mathrm{z}}_{ijklm}=\upmu +{\mathrm{s}}_i+{\mathrm{b}}_{j(i)}+{\mathrm{y}}_{k(i)}+{\upbeta}_l+{\upvarepsilon}_{ijklm} $$

where z_*ijklm*_ is the phenotype of the *m*th individual in the *j*th block in the *k*th year of the *l*th clone from the *i*th site. In Eq. 1, μ denotes the grand mean and ε_*ijklm*_ is the residual term. The clone (β_*l*_, BLUP) and residual term (ε_*ijklm*_) were modeled as random effects, whereas the site (s_*i*_), site/block (b_*j*(*i*)_), and site/year (y_*k*(*i*)_) were treated as fixed effects. The genetic value of each individual was then used as the dependent trait in a univariate linear mixed model for SNP-trait association analyses performed with GEMMA [17]. This method takes relatedness among samples into account through the use of a kinship matrix. The mixed model approach implemented in GEMMA has been shown to outperform methods that try to correct for population structure by including it as a fixed effect in the GWAS analyses [65]. Given the extremely weak population structure we observe in our GWAS population (see “Results”), we did not pursue any further corrections for population structure in the association analyses as this likely would severely reduce our power to detect significant associations. As described previously, we used a FDR < 5% [61] to control for the multiple testing across the 4,425,109 SNPs. We calculated the proportion of variance in phenotype explained by a given SNP (PVE) using the method of Shim et al. [66]:

2 $$ PVE=\frac{2{\widehat{\upbeta}}^2 MAF\left(1- MAF\right)}{2{\widehat{\upbeta}}^2 MAF\left(1- MAF\right)+{\left( se\left(\widehat{\upbeta}\right)\right)}^22 NMAF\left(1- MAF\right)} $$

where $\widehat{\beta}$ and *MAF* is the effect size estimate and minor allele frequency for the SNP, *N* is sample size, and $se\left(\widehat{\beta}\right)$ is standard error of effect size for the SNP.

### Genotype imputation

For some haplotype-based selection tests, imputed and phased datasets were needed. We therefore used BEAGLE v4.1 [67] to perform imputation and haplotype phasing on genotypes of 94 individuals with default parameters. Before performing genotype imputation, we first used Chromosemble from the Satsuma packages [68] to order and orient the scaffolds of the *P. tremula* assembly to 19 pseudo-chromosomes according to synteny with the *P. trichocarpa* genome. We then performed pairwise genome alignment between scaffolds of *P. tremula* and the 19 pseudochromosomes using the BLAST algorithm (*E*-value cut-off of 1e-50) and, finally, > 99% of the SNPs (4,397,537 out of 4,425,109) were anchored on the 19 pseudochromosomes.

To test for the accuracy of imputation, and its relationship with the MAF cutoff and the missing rate of genotypes in our dataset, we selected 346,821 SNPs with a rate of missing genotypes < 10% from the pseudo-chromosome 2 (~ 32.6 Mb) for the simulation analysis. We randomly masked out varying proportions (5–50%) of SNPs, which were treated as missing. BEAGLE v 4.1 was then used to impute genotypes at the masked positions. We found high imputation accuracy (> 0.97) across a wide range of MAF when rates of missing genotypes were < 30% (Additional file 3: Figure S17), suggesting imputation and phasing by BEAGLE should not bias the accuracy of our results. We therefore phased and imputed genotypes of the SNPs anchored on pseudo-chromosomes using BEAGLE v 4.1.

### Estimation of ancestral states for all SNPs

Since the ancestral states of SNPs are usually used for selection detection, for each SNP, we classified alleles as either ancestral or derived on the basis of comparisons with two outgroup species: *P. tremuloides* and *P. trichocarpa*. We obtained publicly available short read Illumina data for one *P. tremuloides* (SRA ID: SRR2749867) and one *P. trichocarpa* (SRA ID: SRR1571343) individual from the NCBI Sequence Read Archive (SRA) [69]. We individually aligned the reads from these two samples to the de novo *P. tremula* assembly (Potra v1.1, available at PopGenIE.org) and used UnifiedGenotyper in GATK to call SNPs at all sites (−-output_mode EMIT_ALL_SITES). For each SNP, two procedures were performed to define their ancestral states: (1) because *P. trichocarpa* is more distantly related to *P. tremula* compared to *P. tremuloides* [70] and from our previous study there were < 1% polymorphic sites shared between *P. tremula* and *P. trichocarpa* [69], we inferred the ancestral state as the *P. trichocarpa* allele at sites where the *P. trichocarpa* individual was homozygous and matched one of the *P. tremula* alleles; otherwise, (2) we inferred the ancestral state as the *P. tremuloides* allele at sites where the *P. tremuloides* individual was homozygous and matched one of the *P. tremula* alleles. If the above two requirements were not met, the ancestral state was defined as missing. In total, we obtained information of ancestral states for 96.3% of all SNPs.

### Anchoring and orientation of SNPs associated with local adaptation to a single region on chromosome 10

As we found that a large majority of significant SNPs (> 90%) detected by at least two of the three methods (PCAdapt, LFMM, and GEMMA) were clustered in a single genomic region on pseudo-chromosome 10, we performed several further steps to refine the anchoring and orientation of these SNPs within this region. First, we used ColaAlignSatsuma from the Satsuma packages [68] to align the genomes of *P. tremula* and *P. trichocarpa* using default settings. The output was then converted and filtered into GBrowse synteny compatible format that was available at http://popgenie.org [50]. Based on the alignment of the two genomes, 15 scaffolds from the *P. tremula* assembly that contain SNPs inferred to be associated with local adaptation were completely or partially mapped to a single region on chromosome 10 of *P. trichocarpa* genome (Additional file 4: Table S3). We then retained only seven scaffolds that were completely mapped to the region and with length > 10 kbp. The seven scaffolds contained > 95% (1465 out of 1528) of the total number of significant SNPs in the single region of chromosome 10. Lastly, according to the alignment results between the genome of *P. tremula* and *P. trichocarpa*, we re-ordered and re-oriented the seven scaffolds to a ~ 700-kbp region for all downstream selection tests (Additional file 3: Figure S4).

### Linkage disequilibrium

To explore and compare patterns of LD between the ~ 700-kbp region on chromosome 10 and genome-wide levels, we first calculated correlations (D’ and *r*^2^) between all pairwise common SNPs (MAF > 5%, 9149 SNPs) in the ~ 700-kbp region using PLINK 1.9 [56]. Then we used PLINK 1.9 to randomly thin the number of common SNPs across the genome to 200,000 and calculated the squared correlation coefficients (*r*^2^) between all pairs of SNPs that were within a distance of 100 kbp. The decay of LD against physical distance was estimated using non-linear regression of pairwise *r*^2^ vs the physical distance between sites in base pairs [71].

### Fine-mapping the causal variants using CAVIAR

We utilized CAVIAR (CAusal Variants Identification in Associated Regions, v1.0) [21] to identify the potential causal variants in the ~ 700-kbp region on chromosome 10. CAVIAR is a fine-mapping method that quantifies the probability of each variant in a locus to be causal and outputs a set of variants that with a predefined probability (e.g. 95% or 99%) contain all of causal variants at the locus. We created the LD structure by computing *r*^2^ between all pairwise significantly associated SNPs in the ~ 700-kbp region using PLINK 1.9. Marginal statistics for each significantly associated variant is the association statistics obtained from GWAS analysis by GEMMA. In our analysis, we set the causal confidence as 99% (−r 0.99) to obtain a set of causal variants that capture all the causal variants with the probability > 99%.

### Positive selection detection

We measured two haplotype-based tests, integrated haplotype score (iHS) [22] and the number of segregating sites by length (nS_L_) [23], to test for possible positive selection. These statistics were calculated for all SNPs with MAF > 0.05 and with information on ancestral state across the genome using the software selscan v1.1.0a [72] with its assumed default parameters. The iHS and the nS_L_ values were then normalized in frequency bins across the whole genome (we used 100 bins). To test for whether there is significant concentration of selection signals on the region surrounding the *PtFT2*, we divided the 19 pseudo-chromosomes (without the seven scaffolds around the *PtFT2* locus) into non-overlapping windows of 700 kbp and calculated the proportion of SNPs with |iHS| > 2 or with |nS_L_| > 2 in each window. Statistical significance was assessed using the ranking of genome-wide windows, with windows having < 100 SNPs being excluded.

### Population-specific selective sweeps

Several standard methods were further applied to search for signs of selective sweeps in different groups of populations: (1) pairwise nucleotide diversity (π) [73], which is expected to have a local reduction following a selective sweep, was calculated using a sliding window approach with window size of 10 kbp and moving step of 5 kbp using the software package - Analysis of Next-Generation Sequencing Data (ANGSD v0.602) [74] separately for South (pop 1-6), Mid (pop 7-8) and North (pop 9-12) populations. Only the reads with mapping quality > 30 and the bases with quality score > 20 were used in the analysis. Windows with < 10% of covered sites remaining from the previous filtering steps (section 2.1) were excluded; (2) Weir and Cockerham’s *F*_ST_, which measures genetic divergence between pairs of three groups of populations, South, Mid, and North, was calculated using a sliding-window approach with window size of 10 kbp and moving step of 5 kbp by VCFtools; (3) a combination of H12 and H2/H1 [27], which measures haplotype homozygosity and can distinguish hard from soft selective sweeps, was calculated in windows of 200 SNPs (~ 15 kbp) for common SNPs with MAF > 5% separately for South, Mid, and North populations. As the mean LD (*r*^2^) in *P. tremula* decays to < 0.1 within 10 kbp (Additional file 3: Figure S11a and [69]), the use of ~ 15 kbp windows should be large enough to differentiate the footprint of selective sweeps from those caused by neutral processes. The H12 and H2/H1 values were then averaged using a sliding window method with window size of 10 kbp and moving step of 5 kbp; (4) a composite likelihood ratio statistic (CLR) [75], which contrasts the likelihood of the null hypothesis based on the genome-wide site frequency spectrum with the likelihood of a model where the site frequency has been altered by a recent selective sweep, was computed using SweepFinder2 [76] separately for South, Mid, and North populations. SweepFinder2 is most efficient when information on the ancestral and derived states is available for SNPs and we therefore polarized SNPs as described above. The small fraction of SNPs (~ 3.7%) that could not be polarized was excluded from further analysis using SweepFinder2. CLRs were calculated using non-overlapping windows with a spacing of 2 kbp; the empirical site frequency spectrum across the whole *P. tremula* genome was estimated using the –f option in SweepFinder2 after including all polymorphic sites in the genome (a total of 8,007,303 SNPs). As recommended by Huber et al. [77], we only used sites that were polymorphic or that represented fixed substitutions in each group of populations to scan for sweeps. To determine whether there are significant differences of the above statistics between the 700-kbp region around *PtFT2* gene on chromosome 10 and genome-wide estimates, we use the same strategy to divide the genome into the windows with the same size for each test and calculated the above statistics across the genome (results are shown in Fig. 4b, d, f, h, j and Additional file 5: Table S4). Significance for the above statistical measurements was evaluated using Mann–Whitney tests.

To assess the scale of a genomic region that is affected by a selective sweep, we ran coalescent simulations modeling a selective sweep in the Northern populations. Simulations were run assuming that the selected site was located at the center of the simulated region. Parameters for the simulations were taken from ABC calculations dating the selective sweep inferred in the North populations (as shown below). Briefly, we used a scaled population mutation rate (4N_e_μ) of 0.0081/bp, which corresponds to the average observed diversity in the North populations. Similarly, we set the scaled population recombination rate (4N_e_r) to 0.0019 to match the genome-wide ratio of r/μ = 0.229 in *P. tremula* [69]. Analyses of the simulated data using SweepFinder2 showed that a single selective sweep often yields multiple significant peaks across a region spanning up to, and even exceeding, 100 kbp (95% quartile: 148,221 bp; Additional file 3: Figure S18).

### Dating the selective sweep in the North populations

To date the inferred selective sweep in the North populations, we used the ABC method described in Ormond et al. [29] to jointly estimate *s* (the strength of selection on the beneficial mutation causing the sweep) and *T* (the time since the beneficial allele fixed) assuming a model of selection from a de novo mutation (hard selective sweep). We simulated 5 × 10^5^ independent selective sweep events using the coalescent simulation program msms [78]. For the coalescent simulations, the ancestries of samples were traced backwards in time using standard coalescent methods and allowing for recombination. Selection was modelled at a single site by applying forward simulations, assuming additive selection so that the fitness of heterozygous and homozygous genotypes carrying the selected (derived) allele were 1 + s/2 and 1 + s, respectively. We simulated a chromosome region consisting of L = 25,000 sites and assumed a diploid effective population size of N_e_ = 92,000, a mutation rate of μ = 3.75 × 10^−8^ per base pair per generation [79], and a recombination rate of *r* = 0.729 × 10^−8^ per base pair per generation. Together these parameters yielded a scaled population mutation rate equal to Θ = 4N_e_μL = 86.27 and a scaled population recombination rate ρ = 4N_e_rL = 19.76. For each simulation, values for both *s* and *T* were drawn from uniform prior distributions, log_10_(T)~U(− 4,– 0.5) and log_10_(s)~U(− 4,– 0.5).

### Gene expression of PtFT2 under active growth and during growth cessation

Samples used for the expression analysis of *PtFT2* were collected from both climate chamber and the field (Sävar, 63.4 °N, Umeå) conditions. For treatment in the climate chamber, two southern clones (SwAsp018, 56.2 °N, Ronneby; SwAsp023, 56.2 °N, Ronneby) and two northern clones (SwAsp100, 63.9 °N, Umeå; SwAsp112, 65.6 °N, Luleå) were selected. These plants were selected to represent the northern-most and southern-most populations of the SwAsp collection that are experiencing the most diverged photoperiodic conditions. Plants were grown under 23-h day lengths for one month and then transferred to 19-h day-length conditions for two weeks before the start of sampling. Leaves were harvested at 2-h intervals for a total period of 24 h using three biological replicates of each genotype. Samples were subsequently flash-frozen in liquid nitrogen and stored at − 80 °C until sample preparation.

Field samples were collected in the Sävar common garden in early July 2014 and samples were taken from two southern clones (SwAsp005, 56.7 °N, Simlång; SwAsp023, 56.2 °N, Ronneby) and two northern clones (SwAsp100, 63.9 °N, Umeå; SwAsp116, 65.6 °N, Luleå). Leaves were harvested from three different clonal replicates planted in the common garden to serve as biological repeats. Leaf samples were flash-frozen in in liquid nitrogen and stored at − 80 °C until sample preparation. Samples were collected at 2-h intervals for a total period of 24 h.

RNA extraction for all samples was performed using a CTAB-LiCl method [80]. Complementary DNA (cDNA) synthesis was performed using the iScript cDNA Synthesis Kit (BIO-RAD) according to the manufacturer’s instructions. Quantitative real-time PCR analyses were performed using a Roche LightCycler 480 II instrument, and the measurements were obtained using the relative quantification method [81]. We used primers qFT2F (5’-AGCCCAAGGCCTACAGCAGGAA-3′) and qFT2R (5’-GGGAATCTTTCTCTCATGAT-3′) for amplifying the transcript of *FT2* and qUBQF (5’-GTTGATTTTTGCTGGGAAGC-3′) and qUBQR (5’-GATCTTGGCCTTCACGTTGT-3′) for *UBQ* as the internal control. We assessed the presence of transcription of both *PtFT*2 (Potra001246g10694) and *PtFT*2β by digesting RT-PCR products with *Sac*I that distinguish the two transcripts (Additional file 3: Figure S7).

### Field experiment with transgenic PtFT2 lines

Construction of the *PtFT* RNAi lines are described in detail in [19]. Briefly, the clone used for transformations is a hybrid aspen, *P. tremula* × *tremuloides*, clone T89, that sets bud at 15-h day lengths [19] and this clone thus has a photoperiodic response that is comparable to SwAsp genotypes from southern Sweden [82]. Transformed T89 plants were planted together with wild type T89 (WT) controls in a common garden at Våxtorp, Halland (latitude 56.4 N, longitude 13.1E) in 2014. Eighteen replicates of each line were planted in a complete randomized block design together with six WT controls per block. Starting in 2015, data were collected on growth cessation, bud formation, and bud set for all trees in the common garden. From early August, plants were visually inspected roughly every five days and top shoots were scored according to a pre-determined scoring sheet (Additional file 3: Figure S19) and classified as active growth (score 3), growth cessation (score 2), bud formation (score 1), and bud set (score 0). Scoring was continued until all plants had completely senesced in late October. Bud scoring data were converted to Julian date of bud set and analyzed using the following linear model:

$$ y_{ij}=\mu + \alpha_i + \beta_j+\varepsilon_{ij} $$

where μ is an overall mean, α_i_ is the effect of treatment *i* (where *i* is either *PtFT*2 RNAi or WT), and β_j_ is the effect of block *j* and ε_ij_ are individual residual errors.

## Additional files

Additional file 1: **Table S1.** Geographical details of the 94 *P. tremula* samples used in this study and the summary statistics of Illumina re-sequencing data per sample. (DOCX 155 kb)

Additional file 2: **Table S2.**. Tracy-Widom statistics for the first three eigenvalues in PCA. (DOCX 31 kb)

Additional file 3: **Figures S1–S19.** (PDF 7335 kb)

Additional file 4: **Table S3.** List of the 1615 candidate SNPs associated with local adaptation. (XLSX 234 kb)

Additional file 5: **Table S4.** Summary statistics (median and central 95% range) for five selective sweep measures across the ~ 700-kbp region around *PtFT2* gene on chromosome 10 and genome-wide level. Pairwise nucleotide diversity (π), genetic divergence between groups of populations (*F*_ST_), H12, H2/H1, and composite likelihood ratio (CLR) test are compared for three groups of populations, South (pop 1–6), Mid (pop 7–8), and North (pop 9–12) corresponding to Fig. 4. (DOCX 95 kb)

Additional file 6: **Table S5.** ANOVA tables for analyses of gene expression in greenhouse and common garden experiments. (DOCX 51 kb)

Additional file 7: **Table S6.** Average values of 39 environmental variables over the years 2002–2012 for the original sample location of 94 *P. tremula* individuals used in this study. (XLSX 64 kb)
