# Supplementary material for: A major locus controls local adaptation and adaptive life history variation in a perennial plant
Source: Genome Biol. 2018 Jun 4;19:72. doi: 10.1186/s13059-018-1444-y (PMC5985590; doi:10.1186/s13059-018-1444-y)
Supplement: Supplementary file 1 — Table S1. Geographical details of the 94 P. tremula samples used in this study and the summary statistics of Illumina re-sequencing data per sample. (DOCX 155 kb) [file 13059_2018_1444_MOESM1_ESM.docx]

**Table S1.** Geographical details of the 94 *Populus tremula* samples used in this study and the summary statistics of Illumina re-sequencing data per sample

| **Sample** | **Origin** | **Pop** | **Latitude** | **Longitude** | **Raw bases(Gb)** | **Filtered bases**  **(Gb)** | **Mapping rate (%)** | **Covered genome (%)** | **Mean coverage** |
| --- | --- | --- | --- | --- | --- | --- | --- | --- | --- |
| SwAsp001 | Simlang | Pop1 | 56.6925 | 13.2147 | 21.39 | 12.02 | 98.97 | 88.28 | 30.23 |
| SwAsp003 | Simlang | Pop1 | 56.7075 | 13.2192 | 10.59 | 8.44 | 98.67 | 88.37 | 21.47 |
| SwAsp004 | Simlang | Pop1 | 56.685 | 13.1964 | 18.44 | 15.57 | 98.92 | 88.99 | 39.15 |
| SwAsp005 | Simlang | Pop1 | 56.7158 | 13.3006 | 8.61 | 7.2 | 98.35 | 87.93 | 17.88 |
| SwAsp009 | Simlang | Pop1 | 56.7336 | 13.2881 | 16.95 | 13.73 | 98.95 | 88.85 | 35.12 |
| SwAsp010 | Simlang | Pop1 | 56.7136 | 13.2922 | 20.59 | 16.67 | 98.84 | 89.04 | 42.67 |
| SwAsp011 | Ronneby | Pop2 | 56.3478 | 15.025 | 34.6 | 29.08 | 98.83 | 89.85 | 72.59 |
| SwAsp012 | Ronneby | Pop2 | 56.3311 | 15.04 | 13.96 | 11.06 | 98.54 | 88.73 | 27.33 |
| SwAsp013 | Ronneby | Pop2 | 56.2828 | 15.0464 | 11.51 | 8.82 | 98.71 | 88.33 | 22.76 |
| SwAsp014 | Ronneby | Pop2 | 56.3081 | 15.1269 | 18.48 | 14.46 | 98.71 | 89.02 | 36.96 |
| SwAsp015 | Ronneby | Pop2 | 56.3081 | 15.1411 | 9.59 | 7.12 | 98.69 | 87.87 | 18.77 |
| SwAsp016 | Ronneby | Pop2 | 56.2203 | 15.3303 | 7.2 | 5.43 | 98.74 | 86.89 | 14.52 |
| SwAsp018 | Ronneby | Pop2 | 56.2178 | 15.3628 | 6.21 | 2.92 | 98.32 | 84.75 | 7.69 |
| SwAsp021 | Vargarda | Pop3 | 57.9917 | 12.9119 | 13.8 | 10.73 | 98.77 | 88.52 | 27.68 |
| SwAsp022 | Vargarda | Pop3 | 57.965 | 12.9547 | 10.38 | 8.12 | 98.71 | 88.17 | 21.28 |
| SwAsp023 | Vargarda | Pop3 | 57.9678 | 12.9272 | 9.89 | 8.63 | 98.45 | 87.96 | 21.42 |
| SwAsp024 | Vargarda | Pop3 | 57.9772 | 12.9383 | 6.76 | 5.08 | 98.63 | 87.13 | 13.41 |
| SwAsp025 | Vargarda | Pop3 | 57.9869 | 12.9358 | 13.65 | 10.08 | 98.68 | 88.63 | 25.99 |
| SwAsp026 | Vargarda | Pop3 | 57.9961 | 12.9047 | 17.45 | 12.82 | 98.77 | 88.84 | 31.07 |
| SwAsp028 | Vargarda | Pop3 | 58.0275 | 12.8989 | 6.57 | 4.45 | 98.61 | 86.59 | 11.86 |
| SwAsp029 | Vargarda | Pop3 | 57.9744 | 12.9014 | 6.34 | 4.3 | 98.52 | 86.57 | 11.4 |
| SwAsp030 | Vargarda | Pop3 | 57.9828 | 12.9567 | 15.81 | 11.47 | 98.56 | 88.89 | 29.54 |
| SwAsp032 | Ydre | Pop4 | 57.8511 | 15.3614 | 15.23 | 12 | 98.59 | 88.9 | 30.84 |
| SwAsp033 | Ydre | Pop4 | 57.8281 | 15.3103 | 15.78 | 11.89 | 98.94 | 88.64 | 30.12 |
| SwAsp034 | Ydre | Pop4 | 57.8014 | 15.3086 | 7.46 | 5.55 | 98.77 | 86.97 | 14.65 |
| SwAsp035 | Ydre | Pop4 | 57.8147 | 15.2858 | 14.69 | 11.09 | 98.75 | 88.64 | 28.61 |
| SwAsp036 | Ydre | Pop4 | 57.8044 | 15.2764 | 10.81 | 8.12 | 98.62 | 88.15 | 21.22 |
| SwAsp037 | Ydre | Pop4 | 57.7919 | 15.1683 | 3.85 | 2.62 | 98.35 | 84.15 | 7.14 |
| SwAsp038 | Ydre | Pop4 | 57.7614 | 15.2303 | 16.47 | 13.35 | 92.35 | 90.12 | 29.81 |
| SwAsp039 | Ydre | Pop4 | 57.7436 | 15.2428 | 5.84 | 4.06 | 98.42 | 86.71 | 10.84 |
| SwAsp040 | Ydre | Pop4 | 57.7422 | 15.2539 | 15.39 | 10.58 | 98.56 | 88.73 | 27.21 |
| SwAsp041 | Brunsberg | Pop5 | 59.6294 | 12.9494 | 14.25 | 9.47 | 98.74 | 88.58 | 23.59 |
| SwAsp042 | Brunsberg | Pop5 | 59.6333 | 12.9536 | 13.73 | 8.95 | 98.82 | 89.42 | 21.83 |
| SwAsp043 | Brunsberg | Pop5 | 59.6325 | 12.9558 | 11.26 | 7.17 | 98.76 | 87.7 | 17.93 |
| SwAsp044 | Brunsberg | Pop5 | 59.6286 | 12.945 | 13.5 | 10.64 | 98.50 | 91.08 | 26.05 |
| SwAsp045 | Brunsberg | Pop5 | 59.6425 | 12.9408 | 15.31 | 11.85 | 98.77 | 88.83 | 30.39 |
| SwAsp046 | Brunsberg | Pop5 | 59.6264 | 12.9683 | 20.77 | 16.92 | 97.12 | 90.2 | 40.39 |
| SwAsp047 | Brunsberg | Pop5 | 59.6308 | 12.9608 | 15.74 | 11.83 | 98.76 | 88.85 | 29.48 |
| SwAsp049 | Brunsberg | Pop5 | 59.6319 | 12.9644 | 18.86 | 14.76 | 98.86 | 89 | 37.6 |
| SwAsp050 | Brunsberg | Pop5 | 59.6347 | 12.9644 | 12.99 | 10.09 | 98.51 | 88.95 | 25.53 |
| SwAsp051 | Uppsala | Pop6 | 59.8317 | 17.7458 | 10.67 | 9.39 | 98.55 | 88.28 | 22.7 |
| SwAsp052 | Uppsala | Pop6 | 59.8161 | 17.8289 | 12.26 | 9.66 | 98.72 | 88.18 | 25,00 |
| SwAsp054 | Uppsala | Pop6 | 59.87 | 17.8275 | 17.8 | 14.51 | 98.88 | 88.91 | 37.34 |
| SwAsp055 | Uppsala | Pop6 | 59.8131 | 17.9817 | 17.01 | 13.14 | 98.71 | 88.9 | 33.77 |
| SwAsp056 | Uppsala | Pop6 | 59.8078 | 17.9833 | 17.37 | 14.1 | 98.70 | 90.83 | 35.32 |
| SwAsp057 | Uppsala | Pop6 | 59.7761 | 17.9889 | 18.44 | 14.12 | 98.61 | 89.12 | 36.2 |
| SwAsp058 | Uppsala | Pop6 | 59.7722 | 17.9867 | 19.29 | 15.92 | 92.72 | 90.22 | 32.57 |
| SwAsp059 | Uppsala | Pop6 | 59.7639 | 17.9786 | 15.65 | 12.11 | 98.37 | 89.03 | 30.95 |
| SwAsp060 | Uppsala | Pop6 | 59.7608 | 17.9781 | 14.41 | 10.69 | 98.51 | 88.69 | 27.64 |
| SwAsp061 | Alvdalen | Pop7 | 61.2219 | 14.0556 | 13.05 | 9.64 | 98.62 | 88.45 | 25.01 |
| SwAsp062 | Alvdalen | Pop7 | 61.215 | 14.1736 | 10.1 | 7.95 | 98.42 | 88.31 | 20.64 |
| SwAsp063 | Alvdalen | Pop7 | 61.175 | 14.2047 | 11.81 | 9.86 | 92.17 | 89.48 | 23.53 |
| SwAsp064 | Alvdalen | Pop7 | 61.1483 | 14.1506 | 10.6 | 8.64 | 98.16 | 88.16 | 21.22 |
| SwAsp065 | Alvdalen | Pop7 | 61.2211 | 13.9017 | 7.44 | 4.65 | 81.96 | 84.27 | 6.2 |
| SwAsp066 | Alvdalen | Pop7 | 61.2017 | 13.8089 | 13.5 | 8.9 | 98.38 | 88.66 | 21.75 |
| SwAsp067 | Alvdalen | Pop7 | 61.1978 | 13.8092 | 16.52 | 13.05 | 98.61 | 88.98 | 33.41 |
| SwAsp068 | Alvdalen | Pop7 | 61.3017 | 13.7222 | 17.18 | 13.47 | 98.61 | 88.88 | 34.64 |
| SwAsp069 | Alvdalen | Pop7 | 61.28 | 13.8144 | 13.34 | 10.25 | 98.67 | 88.59 | 26.5 |
| SwAsp070 | Alvdalen | Pop7 | 61.2575 | 14.0725 | 12.12 | 9.61 | 98.65 | 88.78 | 24.39 |
| SwAsp071 | Delsbo | Pop8 | 61.7692 | 16.6933 | 11.64 | 8.83 | 80.04 | 88.15 | 11.52 |
| SwAsp072 | Delsbo | Pop8 | 61.7589 | 16.7864 | 7.88 | 3.37 | 98.36 | 85.3 | 8.37 |
| SwAsp073 | Delsbo | Pop8 | 61.7511 | 16.7794 | 17.63 | 15.3 | 99.03 | 88.82 | 39.39 |
| SwAsp074 | Delsbo | Pop8 | 61.7211 | 16.75 | 16.07 | 14.05 | 99.10 | 88.48 | 36.09 |
| SwAsp076 | Delsbo | Pop8 | 61.7106 | 16.7311 | 23.91 | 21.32 | 98.87 | 89.25 | 54.27 |
| SwAsp077 | Delsbo | Pop8 | 61.6928 | 16.705 | 12.86 | 10.47 | 98.65 | 88.91 | 27.01 |
| SwAsp078 | Delsbo | Pop8 | 61.6925 | 16.67 | 15.61 | 12.54 | 98.64 | 88.89 | 31.89 |
| SwAsp079 | Delsbo | Pop8 | 61.6978 | 16.6408 | 13.99 | 10.95 | 98.85 | 88.42 | 28.07 |
| SwAsp080 | Delsbo | Pop8 | 61.7892 | 16.5383 | 14.74 | 12.36 | 98.80 | 88.63 | 31.91 |
| SwAsp081 | Dorotea | Pop9 | 64.4183 | 16.4208 | 22 | 18.65 | 99.01 | 89.04 | 47.21 |
| SwAsp082 | Dorotea | Pop9 | 64.3953 | 16.4364 | 12.24 | 10.27 | 98.55 | 88.57 | 26.41 |
| SwAsp084 | Dorotea | Pop9 | 64.3742 | 16.4461 | 12.69 | 10.16 | 98.86 | 88.35 | 26.23 |
| SwAsp086 | Dorotea | Pop9 | 64.3486 | 16.4144 | 13.24 | 10.72 | 98.92 | 88.36 | 27.84 |
| SwAsp087 | Dorotea | Pop9 | 64.3406 | 16.3992 | 17.27 | 14.21 | 98.90 | 88.9 | 36.49 |
| SwAsp088 | Dorotea | Pop9 | 64.3358 | 16.3736 | 16.47 | 13.51 | 98.91 | 88.69 | 34.92 |
| SwAsp089 | Dorotea | Pop9 | 64.3303 | 16.3119 | 14.26 | 11.67 | 98.85 | 88.63 | 30.13 |
| SwAsp090 | Dorotea | Pop9 | 64.3572 | 16.6958 | 13.51 | 11.01 | 98.87 | 88.39 | 28.56 |
| SwAsp091 | Umea | Pop10 | 63.9447 | 20.5942 | 12.4 | 10.25 | 98.81 | 88.52 | 26.79 |
| SwAsp092 | Umea | Pop10 | 63.955 | 20.6261 | 17.15 | 14.21 | 98.94 | 88.72 | 36.62 |
| SwAsp093 | Umea | Pop10 | 63.9844 | 20.6131 | 15.27 | 12.55 | 98.96 | 88.47 | 32.47 |
| SwAsp094 | Umea | Pop10 | 63.9669 | 20.6722 | 16.52 | 14.23 | 95.92 | 90.03 | 34.47 |
| SwAsp095 | Umea | Pop10 | 63.9717 | 20.6875 | 9.99 | 7.98 | 98.75 | 88.1 | 20.89 |
| SwAsp096 | Umea | Pop10 | 63.9781 | 20.7056 | 16.99 | 13.66 | 98.97 | 88.74 | 34.88 |
| SwAsp097 | Umea | Pop10 | 63.8831 | 20.5536 | 17.56 | 14.25 | 98.90 | 88.78 | 36.43 |
| SwAsp098 | Umea | Pop10 | 63.8656 | 20.4986 | 15.94 | 14.14 | 98.97 | 88.57 | 36.67 |
| SwAsp103 | Arjeplog | Pop11 | 66.0247 | 18.5742 | 14.59 | 12.76 | 99.07 | 88.46 | 32.93 |
| SwAsp106 | Arjeplog | Pop11 | 66.2317 | 18.6086 | 23.96 | 20.82 | 98.86 | 89.15 | 52.74 |
| SwAsp109 | Arjeplog | Pop11 | 66.3553 | 18.1803 | 30.22 | 27.46 | 98.93 | 89.91 | 68.18 |
| SwAsp110 | Arjeplog | Pop11 | 66.2592 | 18 | 24.49 | 21.6 | 98.95 | 91.07 | 53.68 |
| SwAsp111 | Lulea | Pop12 | 65.6703 | 21.8986 | 20.95 | 18.23 | 98.97 | 88.86 | 46.86 |
| SwAsp112 | Lulea | Pop12 | 65.6608 | 21.9869 | 21.84 | 18.16 | 98.86 | 89.05 | 46.22 |
| SwAsp113 | Lulea | Pop12 | 65.6989 | 22.1175 | 14.96 | 13.16 | 98.71 | 88.52 | 34.32 |
| SwAsp114 | Lulea | Pop12 | 65.5544 | 22.3939 | 22.96 | 20.22 | 98.90 | 88.91 | 50.85 |
| SwAsp115 | Lulea | Pop12 | 65.5497 | 22.3578 | 25.31 | 21.36 | 98.94 | 89.35 | 53.95 |
| SwAsp116 | Lulea | Pop12 | 65.5594 | 22.3708 | 8.83 | 6.86 | 98.34 | 90.17 | 15.99 |
| Mean |  |  |  |  | 14.84 | 11.81 | 98.10 | 88.55 | 29.72 |
