# Supplementary material for: A major locus controls local adaptation and adaptive life history variation in a perennial plant
Source: Genome Biol. 2018 Jun 4;19:72. doi: 10.1186/s13059-018-1444-y (PMC5985590; doi:10.1186/s13059-018-1444-y)
Supplement: Supplementary file 2 — Table S2.. Tracy-Widom statistics for the first three eigenvalues in PCA. (DOCX 31 kb) [file 13059_2018_1444_MOESM2_ESM.docx]

**Table S2**. Tracy-Widom statistics for the first three eigenvalues in PCA analysis

| **Eigenvectors** | **Eigenvalues** | **Twstat** | ***P*-value** |
| --- | --- | --- | --- |
| 1 | 1.21 | 10.836 | 3.654e-12 |
| 2 | 1.12 | -8.692 | 1 |
| 3 | 1.11 | -10.569 | 1 |
