# Supplementary material for: A major locus controls local adaptation and adaptive life history variation in a perennial plant
Source: Genome Biol. 2018 Jun 4;19:72. doi: 10.1186/s13059-018-1444-y (PMC5985590; doi:10.1186/s13059-018-1444-y)
Supplement: Supplementary file 3 — Figures S1–S19. (PDF 7335 kb) [file 13059_2018_1444_MOESM3_ESM.pdf]

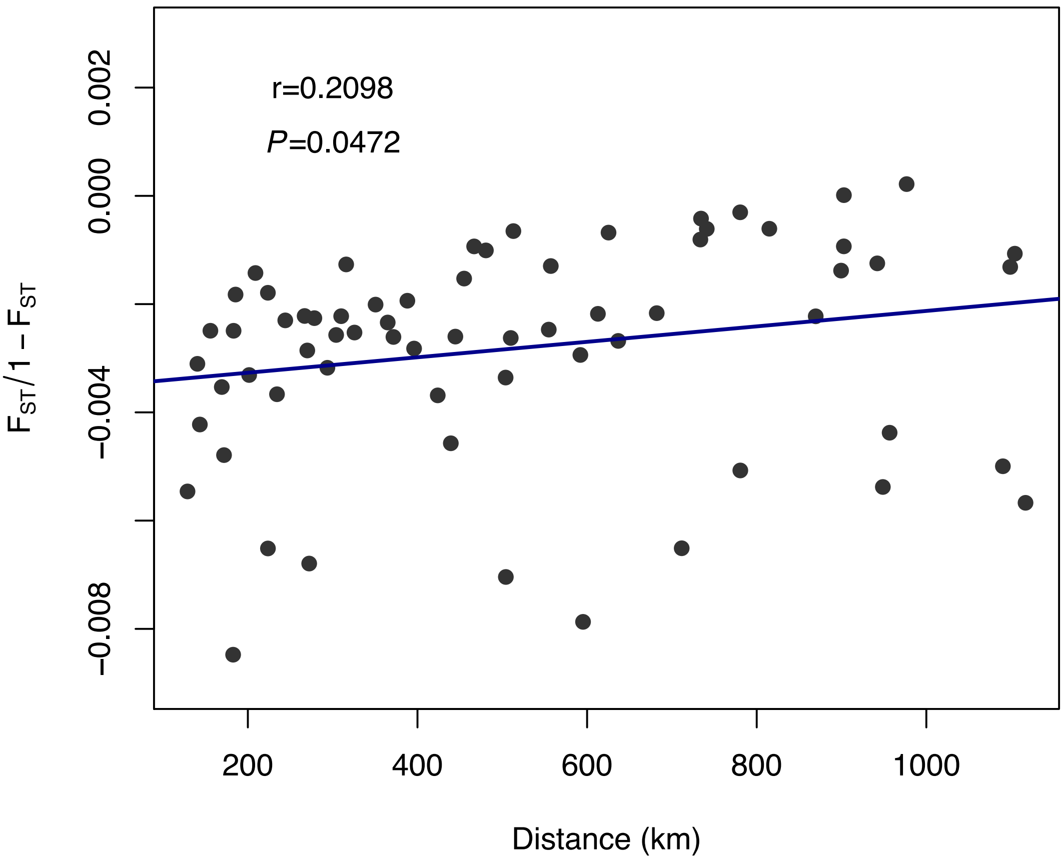

Figure S1 Genetic pairwise differentiation plotted against geographical distances between populations

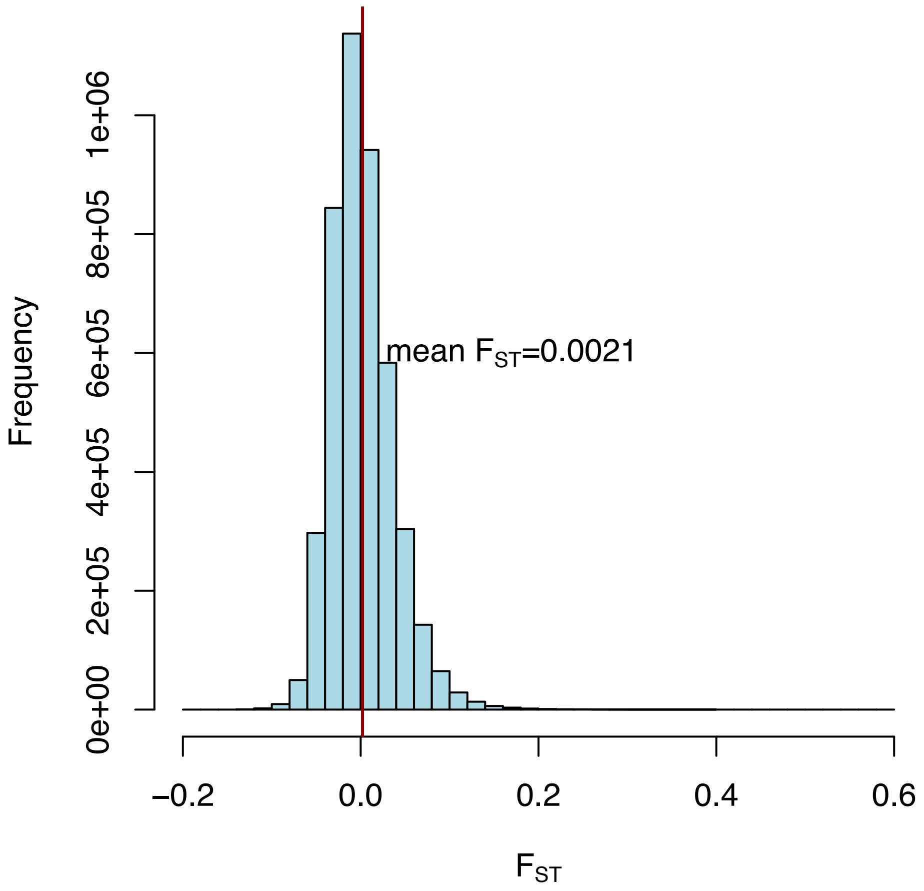

Figure S2 Histogram of Weir & Cockerham's F_{ST} values among the twelve populations.

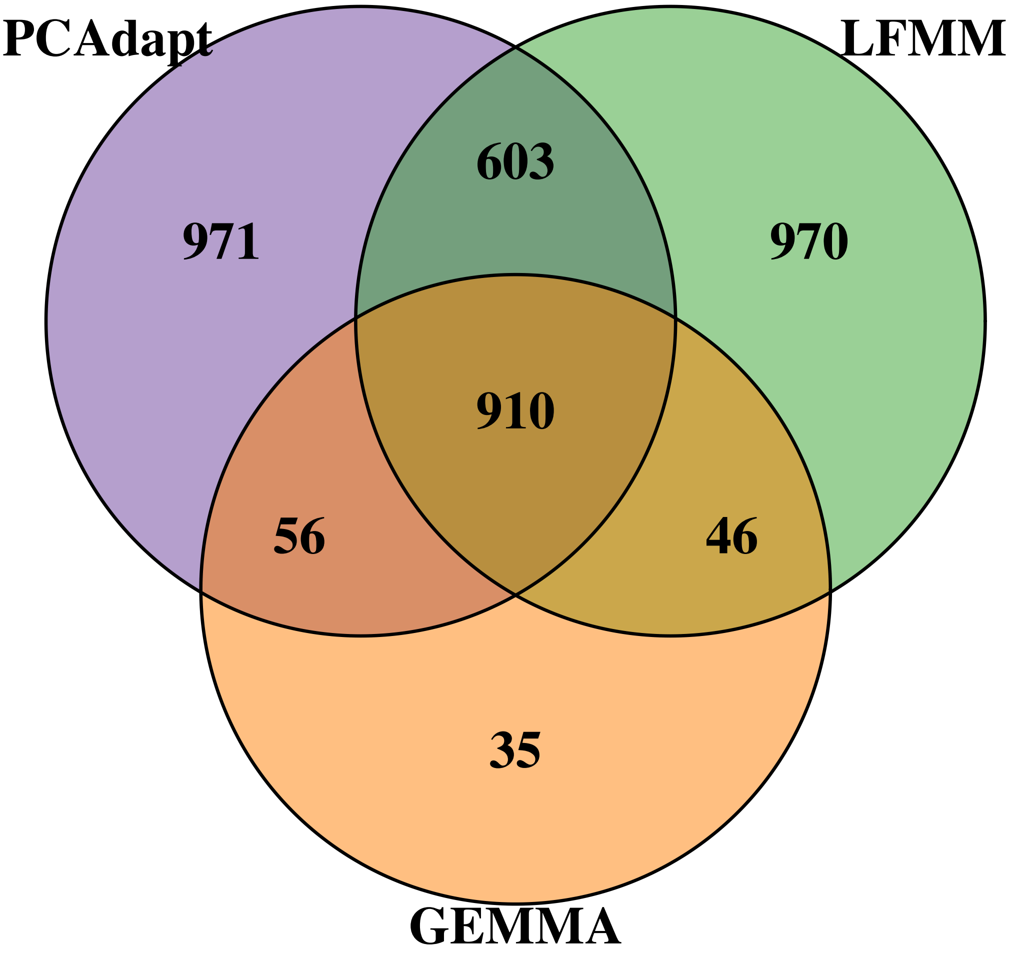

Figure S3 Venn diagram showing the overlap of significant SNPs detected using three approaches of PCAdapt, LFMM and GEMMA.

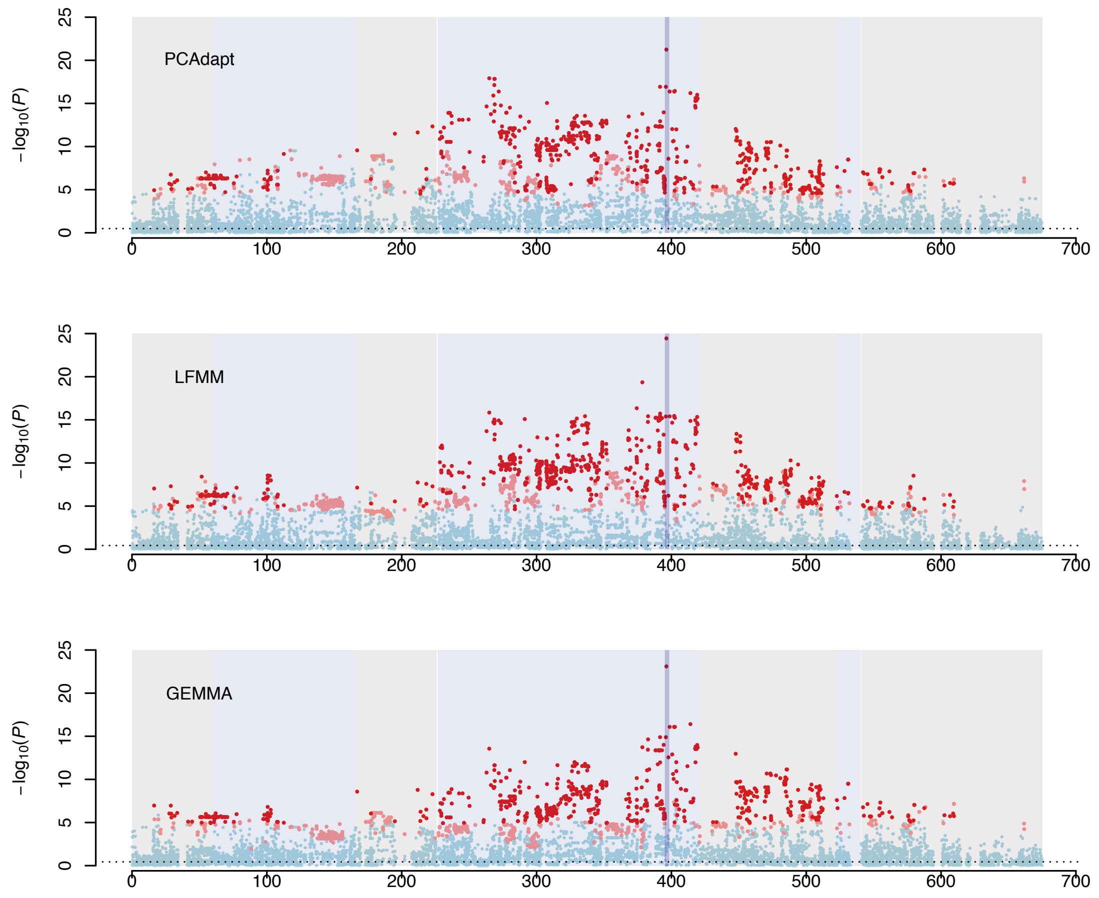

Figure S4 Magnified view of negative $\log_{10}$-transformed *P* values calculated from three approaches, PCAdapt, LFMM and GEMMA (from top to bottom) for all SNPs in the seven adjacent scaffolds (with length > 10 kbp, shown as boxes with alternating shades), which were anchored to a single region (~700 kbp) on chromosome 10 based on the genome alignment between *P. tremula* and *P. trichocarpa*. The significant SNPs (at false discovery rate of *Q*-value <0.05) identified by all three approaches are denoted by red dots, and those identified by two of the three approaches are denoted by pink dots. The light blue dots represent those non-significant SNPs. Dotted horizontal line represents the genome-wide average value of $-\log_{10}(P)$ calculated by each method. The genomic locations of *PtFT2* gene within this region are shaded as a grey bar. The seven scaffolds from left to right are Potra000799, Potra000908, Potra000342, Potra001246, Potra004002, Potra003230, Potra000530, respectively.

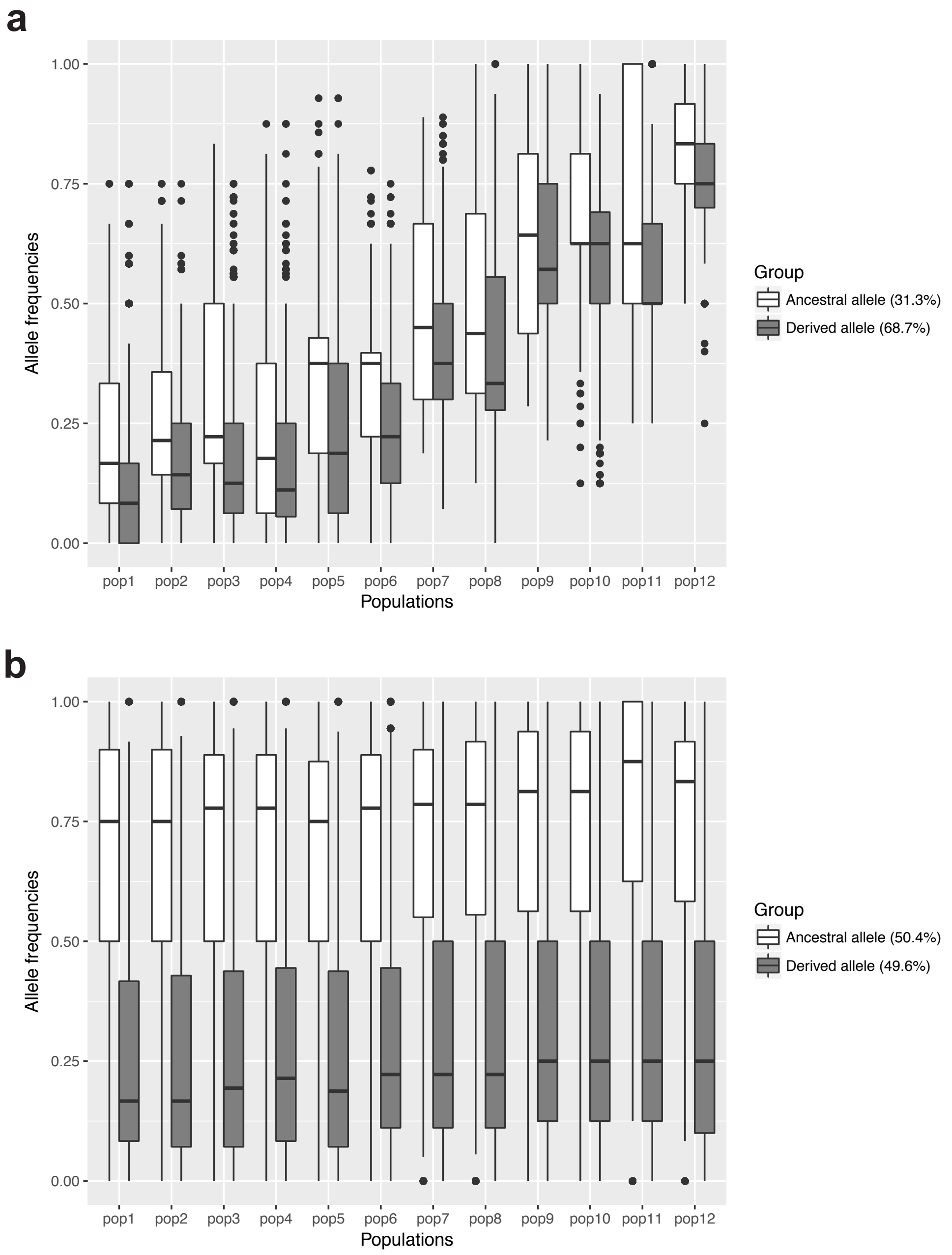

Figure S5 Derived- and ancestral- allele frequency plotted against population for significant SNPs involved in local adaptation on chromosome 10 (a) and for 10,000 randomly selected SNPs from the genome (b). Alleles are polarized according to the signs of the Spearman's rank correlations with the first environmental principal components (PC1), where only the derived (grey boxes) or ancestral (white boxes) allele with a positive correlation with the environmental PC1 is shown for each SNP.

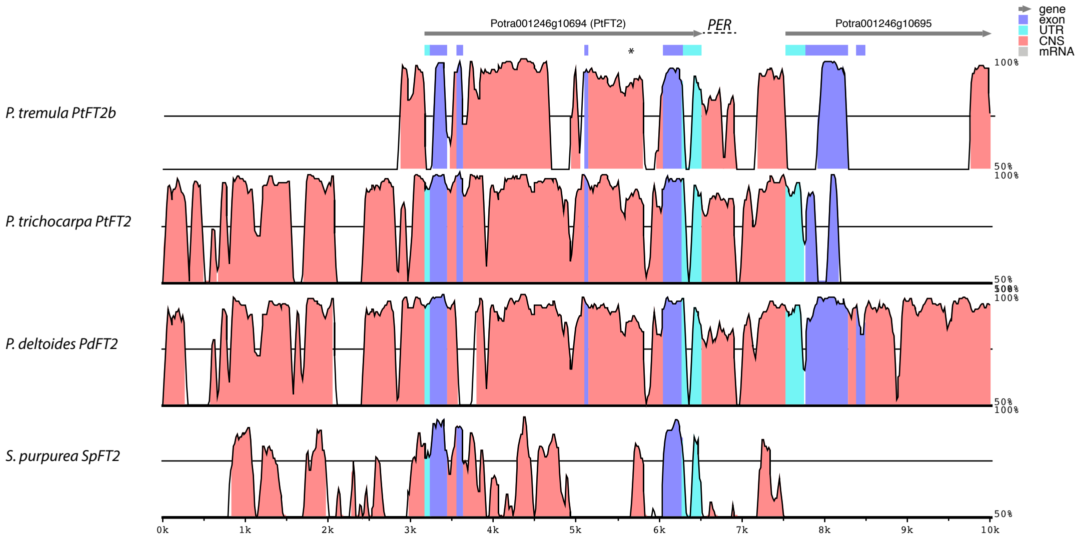

Figure S6 Plot of a pairwise alignment for genome region containing Potra001246g10694 (*PtFT*2) and Potra001246g10695 with *PtFT*2β, and the corresponding genomic region from *P. trichocarpa*, *P. deltoides* and *Salix purpurea*. Curves were calculated using default VISTA thresholds based on percentage identity (y-axis) and base pair position (x-axis), and only the regions longer than 100 bp with average conservation score above threshold of 50% were colored (exons in blue, introns and promoter in pink, and UTR in light blue). A motif scan using PlantCARE (http://bioinformatics.psb.ugent.be/webtools/plantcare/html/) failed to identify any conserved motifs or transcription factor binding sites in the region surrounding the location of the most strongly associated SNP in *PtFT*2 (marked by a * in the figure).

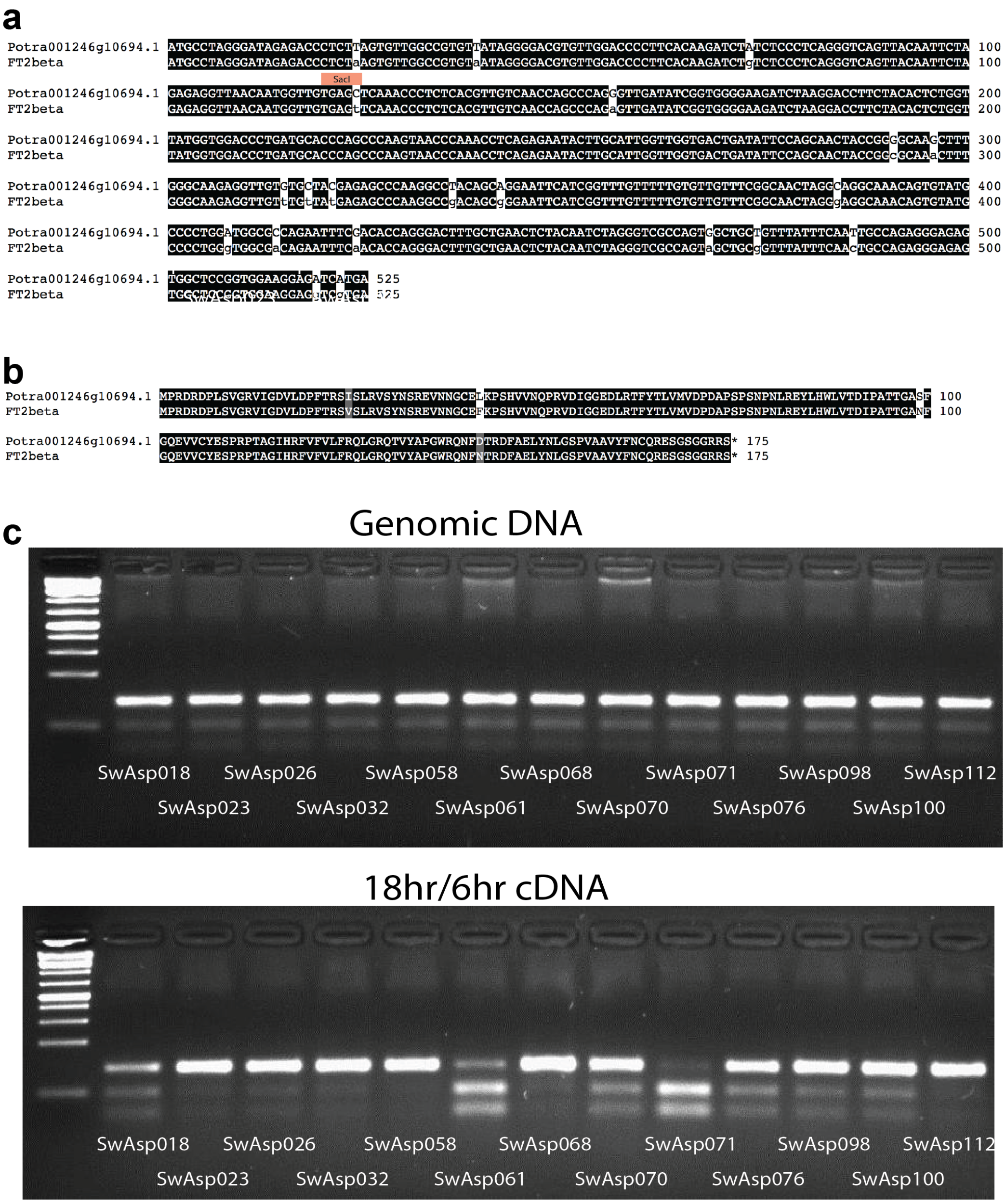

Figure S7 a) Nucleotide alignment between the two copies of *PtFT*2 located on scaffold Potra001246 - Potra001246g10694 and PtFT2β. The red box entitles *Sac*I a CAPS marker that distinguishes the two loci using the presence (Potra001246g10694) or absence (PtFT2β) of a *Sac*I cut site. b) Protein alignment of - Potra001246g19694 and PtFT2β. c) Transcripts of *PtFT*2 and *PtFT*2β are distinguished by the presence /absence of a *Sac*I cut site. Top figure show restriction digests with *Sac*I of PCR products targeting *PtFT*2/*PtFT*2β using genomic DNA as a template. The bottom figures show corresponding PCRs using cDNA taken from plants growing in 18h light and 6 hr dark as a template. There was no evidence for alternative splicing of *PtFT*2 in the RT-PCR experiments.

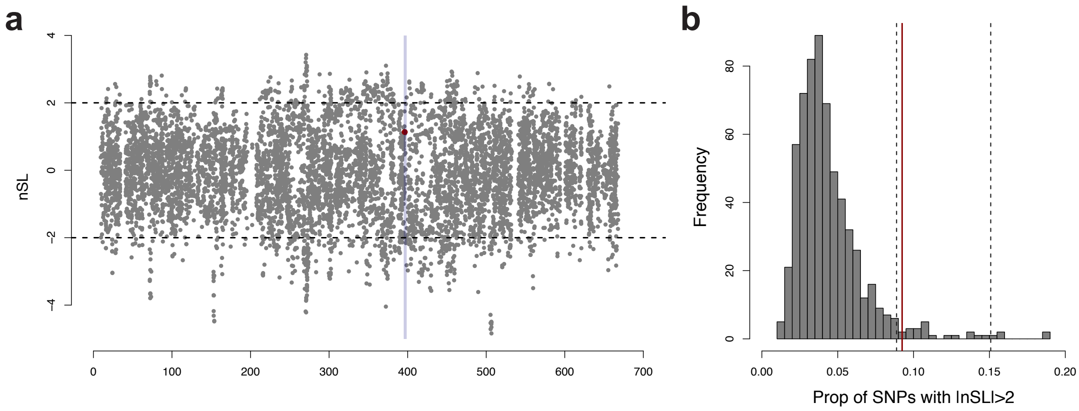

Figure S8 A high concentration of significant nSL signals was found in the ~700 kbp region around *PtFT2* gene. (a) Patterns of normalized nSL scores (y-axes) across the ~700 kbp genomic region (x-axis) around the *PtFT2* gene (shaded as grey bar). The dashed horizontal lines indicate the threshold of positive selection signal (|nSL|>2.0). The red dot indicates the SNP (Potra001246:25256) showing the strongest signal of local adaptation. We then divided the genome into 626 non-overlapping regions with size of 700 kbp (without the candidate region and regions with less than 1000 SNPs left were removed) and calculated the proportion of significant |nSL| SNPs (MAF >5%) that lie in each 700 kbp region. (b) The ~700 kbp region around *PtFT2* gene (the red lines) contained significant (empirical *P*-value <0.05) higher proportion of SNPs with signals of positive selection relative to genome-wide distribution (dark grey bars) (ranked 23th among 627 regions). The dashed lines represent the 95% and 99% quantiles, respectively.

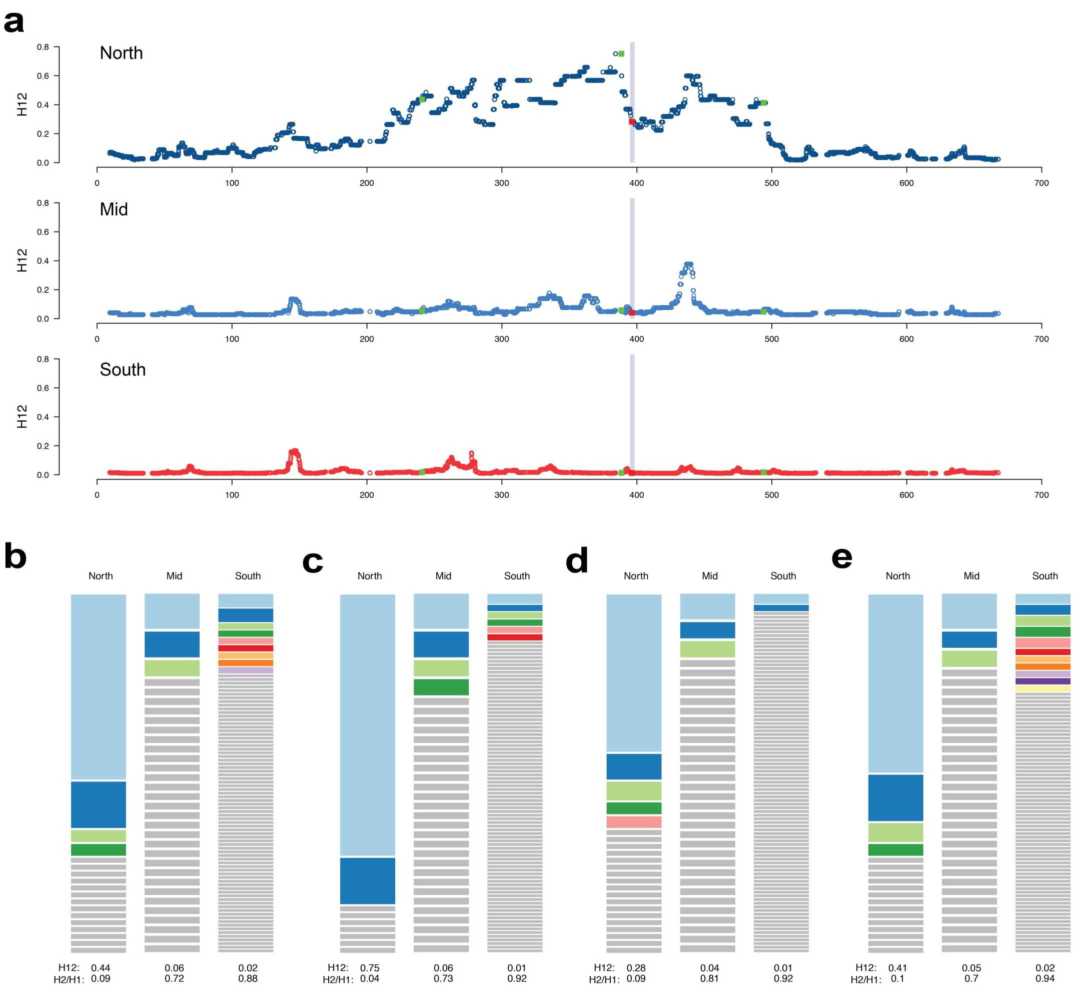

Figure S9 H12 scan for selective sweeps. (a) H12 scan in three groups of populations, South (pop 1-6, bottom), Mid (pop 7-8, middle) and North (pop 9-12, top), across the ~700 kbp region around *PtFT2* gene on chromosome 10. Each data point represents the H12 values calculated at each common SNP (minor allele frequency higher than 5%). The genomic location of *PtFT2* gene within this region is shaded as a grey bar. We picked the SNP (Potra001246:25256, red square) showing the strongest signal of local adaptation and another three randomly selected SNPs (green square) to show the haplotype frequency spectra (b-e) in each group of populations at this region (b-e, corresponding to the locations of the four SNPs from left to right). In each haplotype frequency spectra plot, the height of the first bar (light blue) in each frequency spectrum indicates the frequency of the most prevalent haplotype in each group of samples, and heights of subsequent colored bars indicate the frequency of the second, third and so on most frequent haplotypes in the samples. Grey bars indicate singletons. The values of H12 and H2/H1 are shown at the bottom of each bar plot. In northern populations, there is mainly a single haplotype dominating the haplotype spectra, indicative of hard sweeps with high H12 values but low H2/H1 values. No obvious selective sweep signals were found in either middle or southern populations.

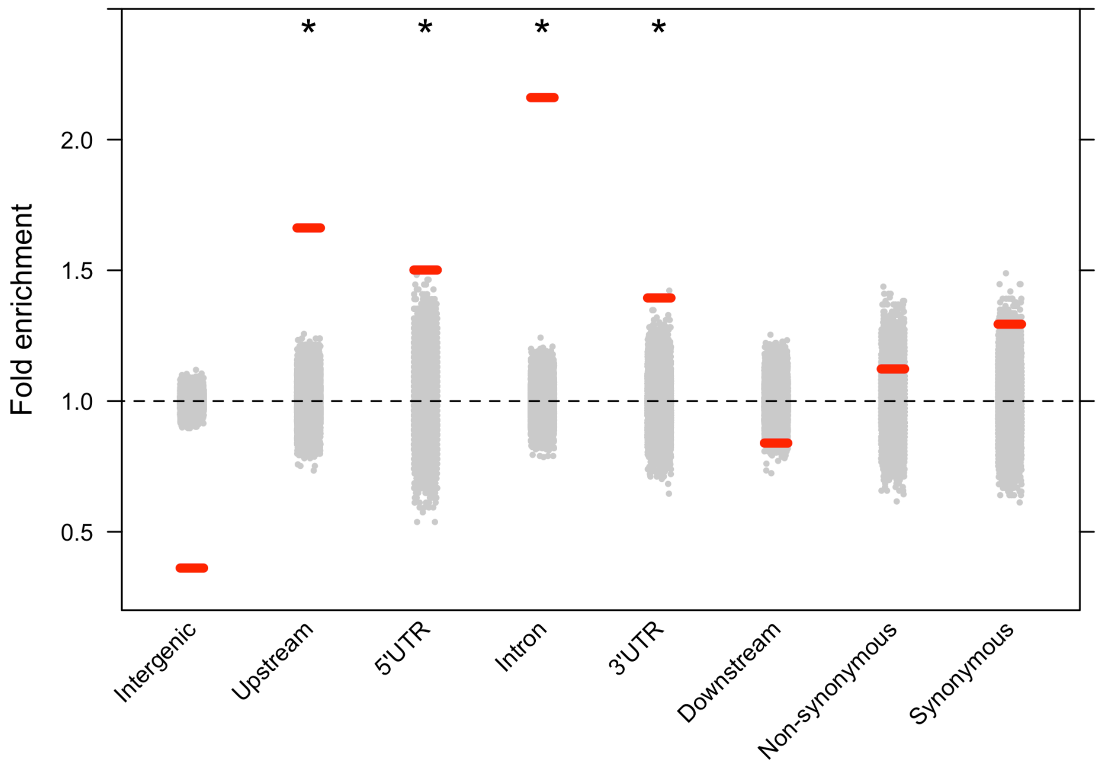

Figure S10 Enrichment of various functional categories in significant SNPs associated with local adaptation (red line). Grey dots show the distribution of results with 10000 bootstrap replicates. The dashed line shows the expected enrichment under the null hypothesis of no enrichment. Enrichments that is significant relative to the bootstrap method are denoted by asterisks (*P*<0.001).

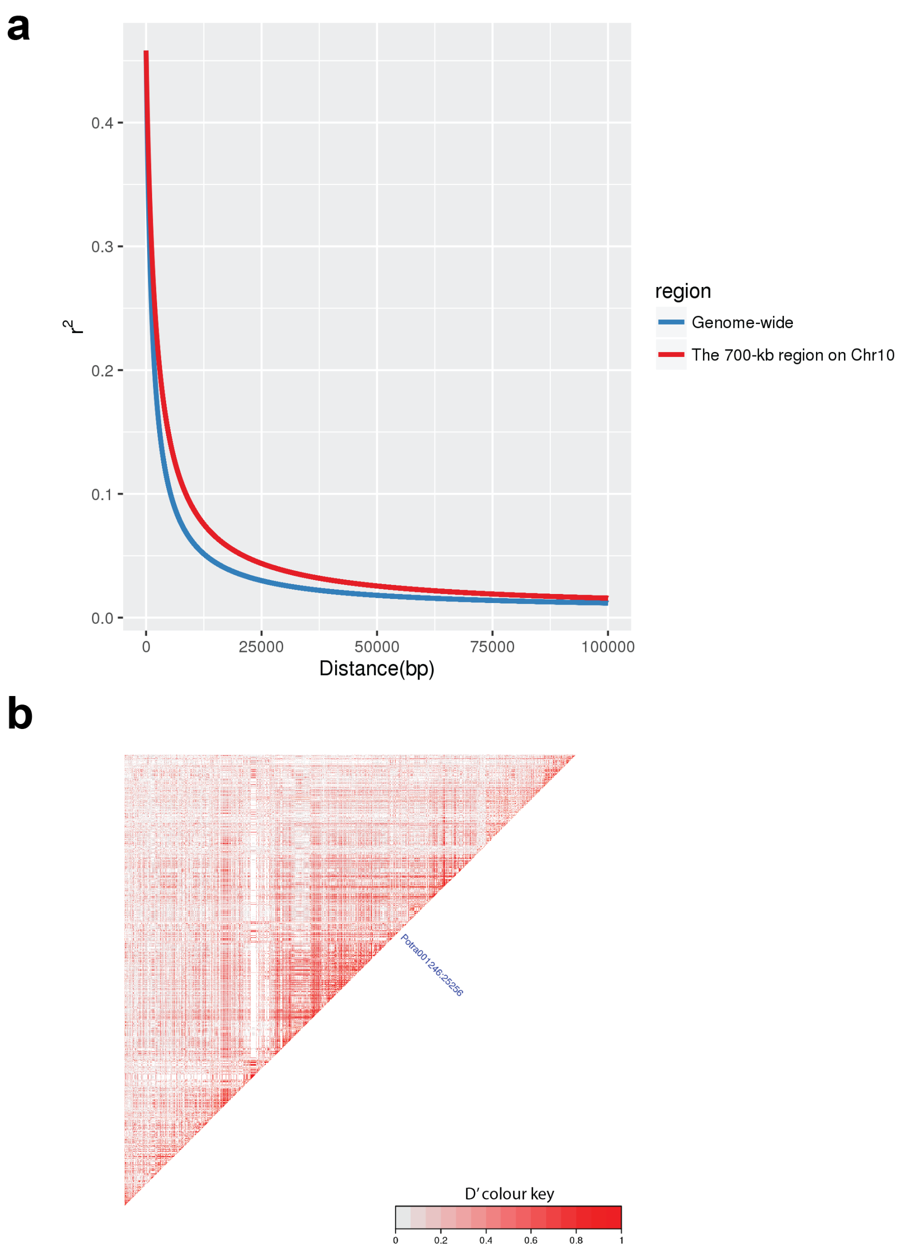

Figure S11 (a) Decay of linkage disequilibrium (LD). The comparison of mean LD decay (estimated as r^2) with physical distance between the ~700 kbp region on chromosome 10 and genome-wide average level. (b) Pairwise linkage disequilibrium (quantified using D') among the 9149 common SNPs (minor allele frequency higher than 5%) within the ~700 kbp region on chromosome 10.

a

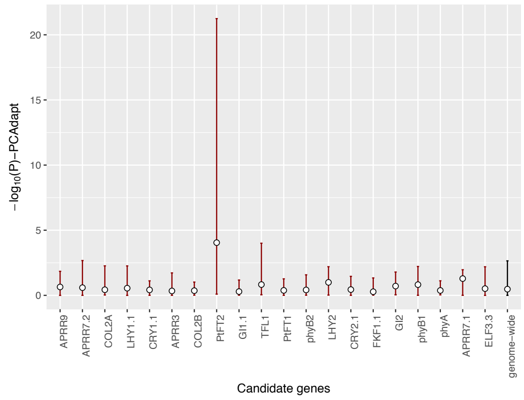

b

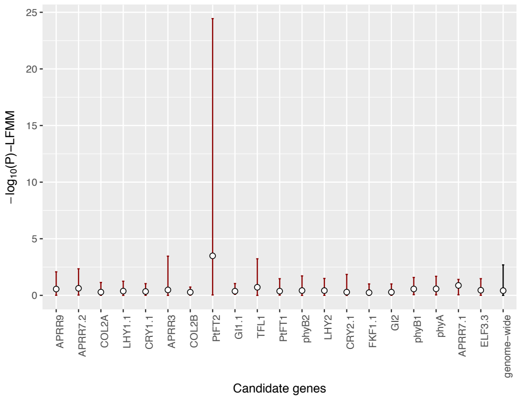

c

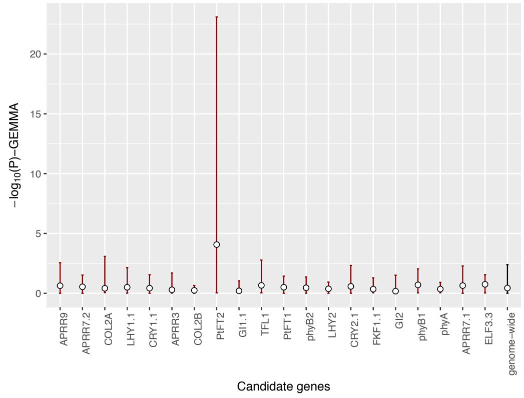

d

Gene	P.trichocarpa v3.0	Aspen v1.1
**APRR9**	Potri.015G061900	Potra000433g02322
**APRR7.2**	Potri.010G215200	Potra000560g04057
**COL2A**	Potri.017G107500	Potra000774g06101
**LHY1.1**	Potri.002G180800	Potra000884g07139
**CRY1.1**	Potri.005G164700	Potra000891g07204
**APRR3**	Potri.015G002300	Potra001064g09139
**COL2B**	Potri.004G108300	Potra001130g09930
**PtFT2**	Potri.010G179700	Potra001246g10694
**GI1.1**	Potri.005G196700	Potra001576g13038
**TFL1**	Potri.004G203900	Potra001614g13295
**PtFT1**	Potri.008G077700	Potra001726g14043
**phyB2**	Potri.010G145900	Potra001834g14751
**LHY2**	Potri.014G106800	Potra001834g14753
**CRY2.1**	Potri.008G166600	Potra001882g14990
**FKF1.1**	Potri.010G105700	Potra002303g17572
**GI2**	Potri.002G064400	Potra002370g18052
**phyB1**	Potri.008G105200	Potra002727g19853
**phyA**	Potri.013G000300	Potra002935g20343
**APRR7.1**	Potri.008G046200	Potra003966g23819
**ELF3.3**	Potri.006G233800	Potra003980g23954

Figure S12 Signatures of local adaptation for a set of 20 candidate genes controlling phenology in *Populus*. (a-c) Distribution of negative $\log_{10}$-transformed *P* values calculated from three approaches, PCAdapt (a), LFMM (b) and GEMMA (c) for common SNPs (minor allele frequency>5%) within the 20 candidate genes (red lines) and all other genes across the genome (black lines). For the 20 candidate genes, the bottom and the top of the error bars represent the lowest and highest negative $\log_{10}$-transformed *P* values of each method. For the rest of genes across the genome, the bottom and the top of the error bars represent 0.5^{th} and 99.5^{th} percentiles negative $\log_{10}$-transformed *P* values. From the results, we found that except for *PtFT2* gene, it is hard to distinguish the signatures of local adaptation of all other candidate genes from the rest of genes across the genome. (d) The list of gene names (corresponding to the *Populus trichocarpa* v3 assembly and *P. tremula* v1.1 assembly) of 20 candidate genes homologous to the *Arabidopsis thaliana* phenology genes shown in a-c.

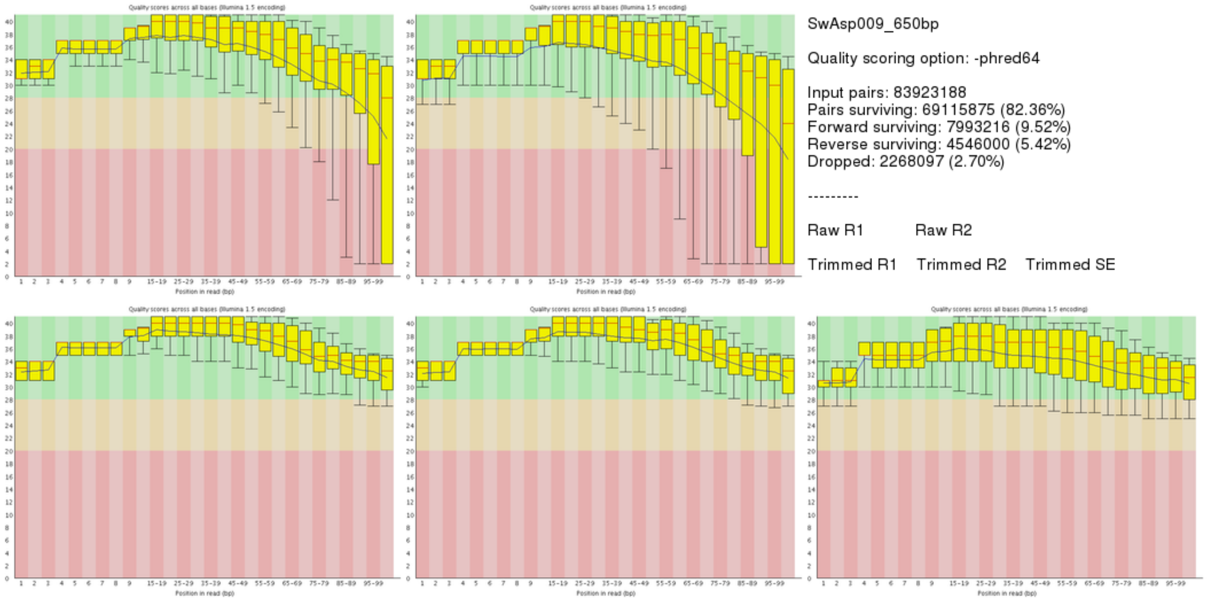

Figure S13 Comparison of per-base sequence quality between raw and filtered sequence data in one SwAsp sample (SwAsp009) as an example. Per-base sequence quality comparison between raw paried-end sequence data (forward reads: top left and reverse reads: top right), and filtered sequence data with both forward (bottom left) and reverse (bottom middle) reads left or only single-end (bottom right) reads left. The x-axis of the BoxWhisker plot shows the position in read, and y-axis shows the quality scores. The higher the score the better the base call. The background of the plot divides the y axis into very good quality calls (green), calls of reasonable quality (orange), and calls of poor quality (red). The central red line is the median quality value, and yellow box represents the inter-quantile of quality, the upper and lower whiskers represent the 10% and 90% points, the blue line represents the mean quality.

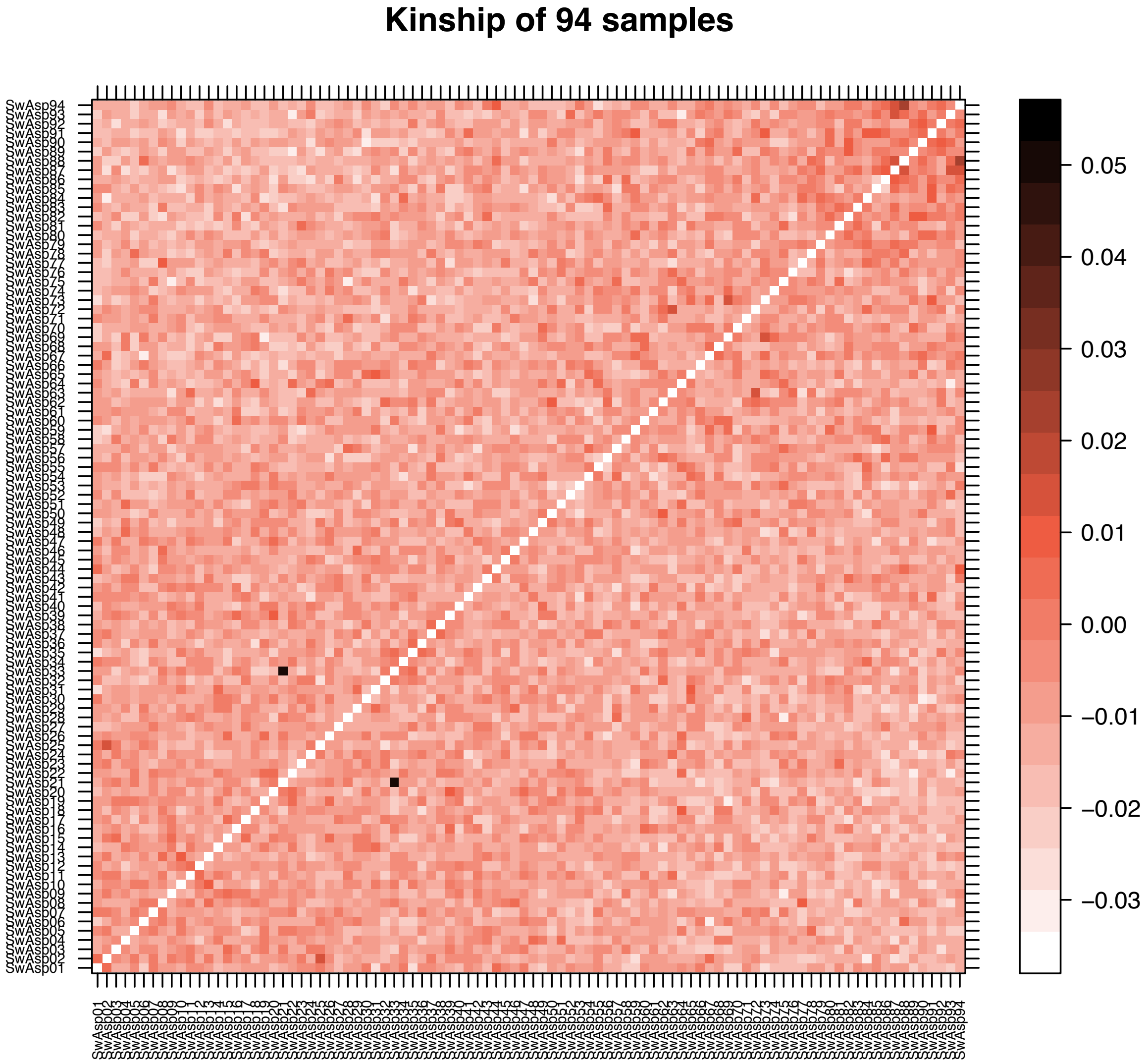

Figure S14 Kinship relationships among the 94 *P. tremula* individuals. The values of the relatedness statistics were calculated according to the method implemented in GEMMA.

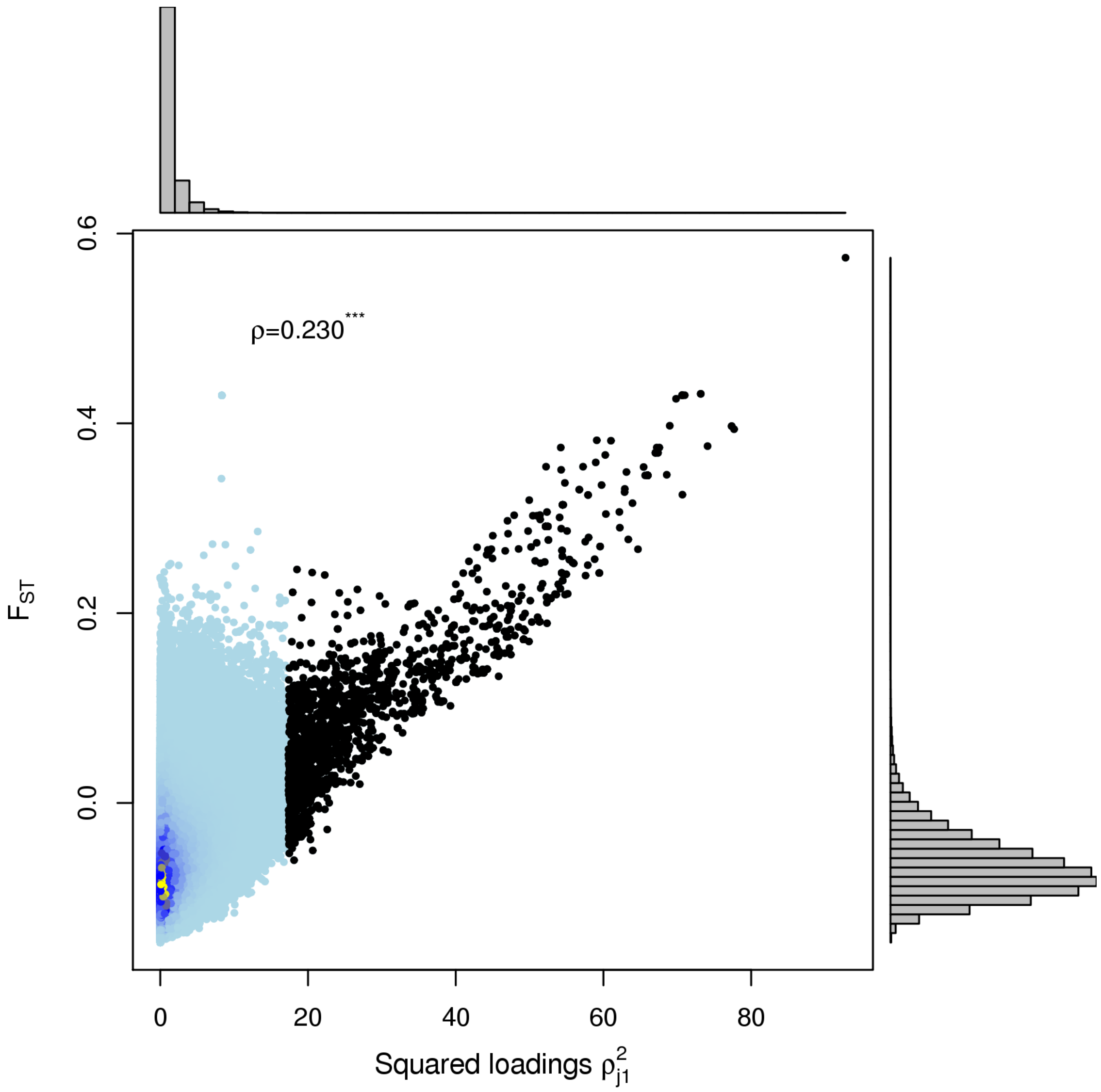

Figure S15 Two-dimensional distribution for squared loadings $\rho^2{}_{j1}$ with the first environmental principal component estimated from PCAdapt and Weir & Cockerham's F_{ST} values of the common SNPs with minor allele frequency higher than 5%. The yellow to dark blue to light blue gradient indicates decreased density of observed events at a given location in the graph. Black dots represent SNPs fulfilling the significance threshold requirement defined by PCAdapt.

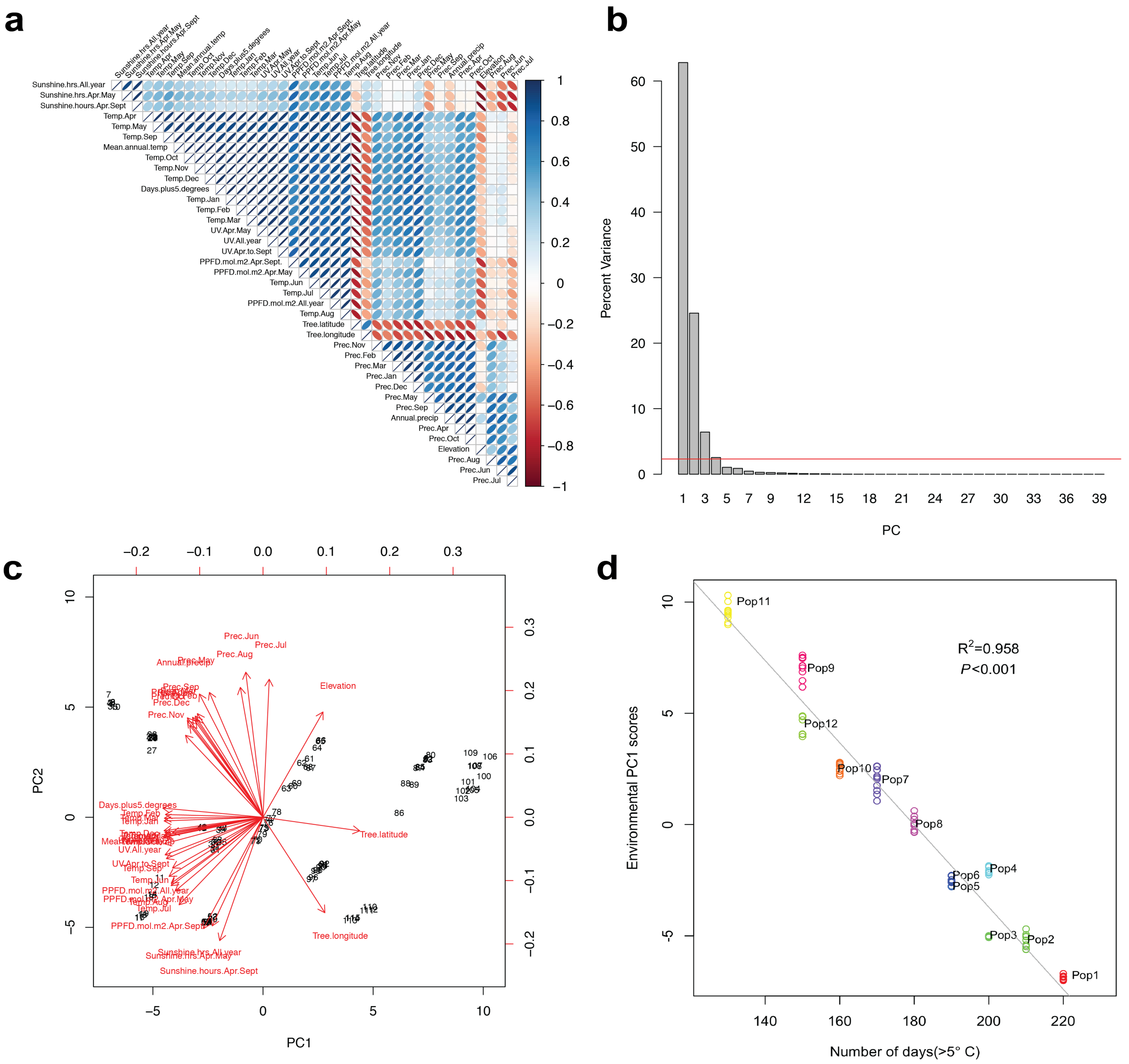

Figure S16 (a) Correlations between pairs of the 39 environmental variables. Blue indicates a positive relationship, and red indicates a negative relationship. Color intensity is proportion to Pearson's correlation coefficient. (b) The percent of explained variance for each principal component (PC) from the principal component analysis (PCA) of all 39 environmental variables. (c) Biplot for all environmental variables loaded on the top two PCs. (d) The relationship between scores of the first environmental principal component (PC) and the length of growing season, which is represented as the number of days with temperature higher than 5 ºC, for samples in the 12 populations of *P. tremula*.

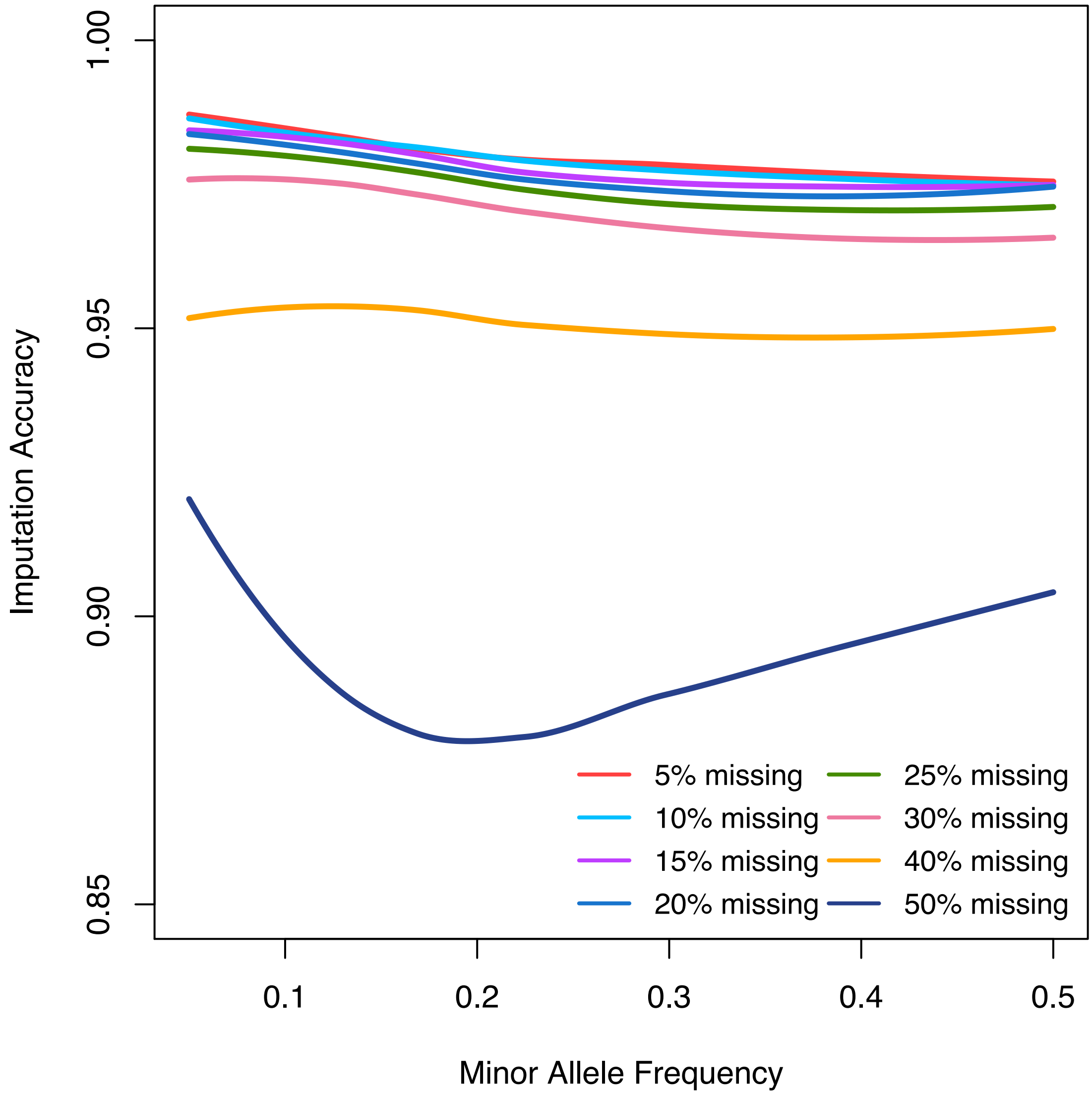

Figure S17 Comparison between imputation accuracy with minor allele frequency (MAF) under a simulation test. Color lines shows imputation accuracy compared with MAF under various ratio of artificial missed SNPs had been imputed by BEAGLE v 4.1.

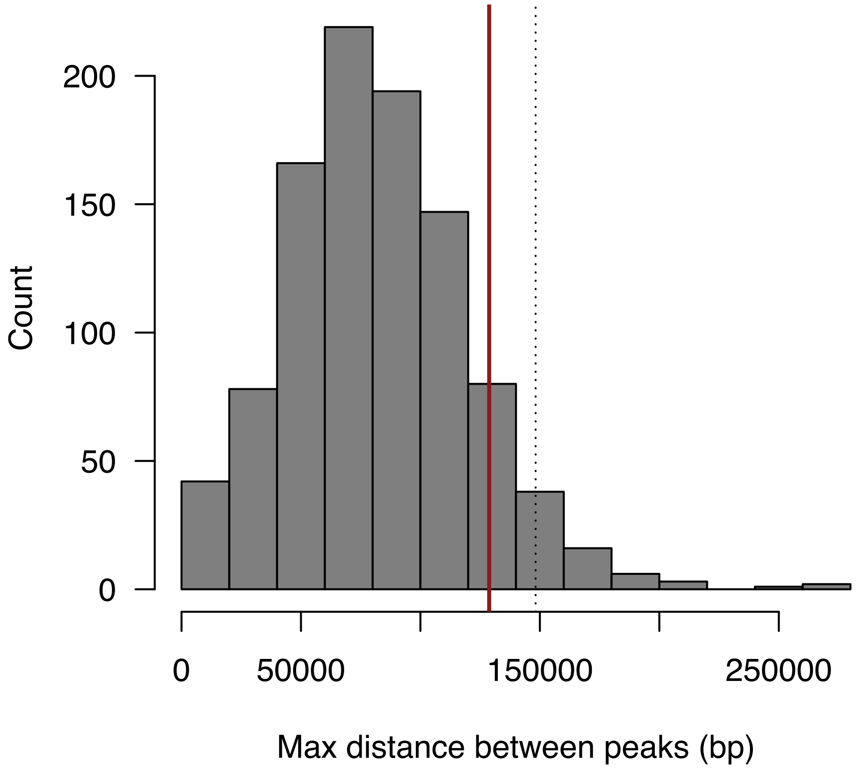

Figure S18 Distribution of maximum distance between significant composite likelihood ratio (CLR) peaks calculated using the simulated data from SweepFinder2. The dotted line denotes the 95% quantile and the dark red line indicates the value calculated from the empirical dataset in this study.

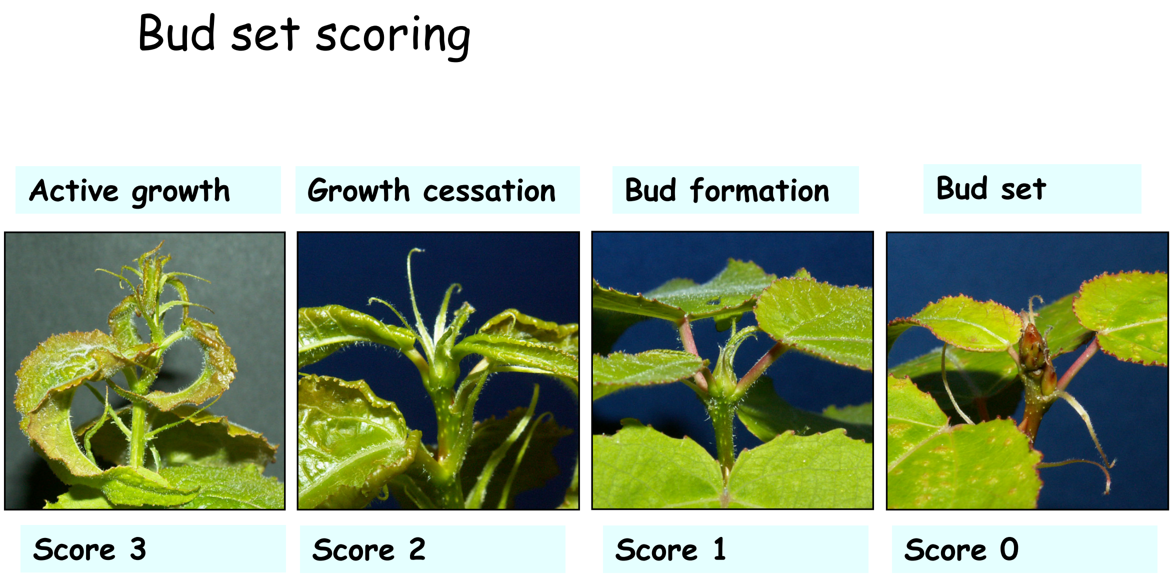

Figure S19 Key used for scoring bud set in the field experiment with transgenic *PtFT2* lines at Våxtorp, Sweden.
