# Supplementary material for: A major locus controls local adaptation and adaptive life history variation in a perennial plant
Source: Genome Biol. 2018 Jun 4;19:72. doi: 10.1186/s13059-018-1444-y (PMC5985590; doi:10.1186/s13059-018-1444-y)
Supplement: Supplementary file 5 — Table S4. Summary statistics (median and central 95% range) for five selective sweep measures across the ~ 700-kbp region around PtFT2 gene on chromosome 10 and genome-wide level. Pairwise nucleotide diversity (π), genetic divergence between groups of populations (FST), H12, H2/H1, and composite likelihood ratio (CLR) test are compared for three groups of populations, South (pop 1–6), Mid (pop 7–8), and North (pop 9–12) corresponding to Fig. 4. (DOCX 95 kb) [file 13059_2018_1444_MOESM5_ESM.docx]

**Table S4.** Summary statistics (median and central 95% range) for five selective sweep measures across the ~700 kb surrounding *PtFT2* locus and across genome wide. Pairwise nucleotide diversity (π), genetic divergence between groups of populations (*F*_ST_), H12, H2/H1 and composite likelihood ratio (CLR) test are compared for three groups of populations, South (pop 1-6), Mid (pop 7-8) and North (pop 9-12) corresponding to Fig. 4.

| **Measures** | **Group** | **Genome-wide** | **Chr10-700kb** |
| --- | --- | --- | --- |
| **π** | North | 0.0129(0.0040-0.0302) | 0.0077(0.0024-0.0157)*** |
|  | Mid | 0.0138(0.0049-0.0305) | 0.0111(0.0047-0.0205)*** |
|  | South | 0.0134(0.0045-0.0300) | 0.0105(0.0037-0.0223)*** |
|  |  |  |  |
| **F_ST_** | N vs. S | -0.0001(-0.0132-0.0361) | 0.1012(0.0076-0.3144)*** |
|  | N vs. M | -0.0028(-0.0221-0.0464) | 0.0258(-0.0129-0.1197)*** |
|  | S vs. M | -0.0021(-0.0179-0.0392) | 0.0218(-0.0038-0.1006)*** |
|  |  |  |  |
| **H12** | North | 0.0266(0.0200-0.1206) | 0.1620(0.01997-0.5991)*** |
|  | Mid | 0.0346(0.0277-0.1177) | 0.0429(0.0277-0.1537)*** |
|  | South | 0.0142(0.0104-0.0820) | 0.0140(0.0104-0.0785) |
|  |  |  |  |
| **H2/H1** | North | 0.8714(0.2455-0.9808) | 0.3125(0.0403-0.9310)*** |
|  | Mid | 0.9000(0.3250-0.9737) | 0.8043(0.2308-0.9737)*** |
|  | South | 0.9135(0.2394-0.9898) | 0.9174(0.1552-0.9898) |
|  |  |  |  |
| **CLR** | North | 0.2736(0-15.6112) | 0.4257(0.0001-115.6340)*** |
|  | Mid | 0.2487(0-17.8958) | 0.1984(0-6.3314) |
|  | South | 0.3340(0-14.7442) | 0.1896(0-6.1393) |

Significant differences between ~700 region around *PtFT2* gene and genome wide estimates are determined by one-sided Mann-Whitney U test for each test.

*** P<0.0001
