# Supplementary material for: A major locus controls local adaptation and adaptive life history variation in a perennial plant
Source: Genome Biol. 2018 Jun 4;19:72. doi: 10.1186/s13059-018-1444-y (PMC5985590; doi:10.1186/s13059-018-1444-y)
Supplement: Supplementary file 6 — Table S5. ANOVA tables for analyses of gene expression in greenhouse and common garden experiments. (DOCX 51 kb) [file 13059_2018_1444_MOESM6_ESM.docx]

**Table S5.** Anova tables for analyses of gene expression in greenhouse and common garden experiments

| **Greenhouse experiment** | |  |  |  |  |
| --- | --- | --- | --- | --- | --- |
|  | df | SumSq | MeanSq | F | p |
| ZT | 19 | 98141 | 5165 | 6,33 | 9,50E-13 |
| Genotype | 3 | 360846 | 120282 | 147,43 | 2,00E-16 |
| Replicate | 8 | 4990 | 624 | 0,76 | 0,6344 |
| Residuals | 208 | 169699 | 816 |  |  |
| **Common garden experiment** | |  |  |  |  |
|  | df | SumSq | MeanSq | F | p |
| ZT | 11 | 578,36 | 52,579 | 9,33 | 3,06E-12 |
| Genotype | 3 | 936,21 | 312,069 | 55,36 | 2,20E-16 |
| Replicate | 8 | 574,92 | 71,865 | 12,75 | 1,79E-13 |
| Residuals | 132 | 744,03 | 5,637 |  |  |
